# Bcor loss perturbs myeloid differentiation and promotes leukaemogenesis

**DOI:** 10.1038/s41467-019-09250-6

**Published:** 2019-03-22

**Authors:** Madison J. Kelly, Joan So, Amy J. Rogers, Gareth Gregory, Jason Li, Magnus Zethoven, Micah D. Gearhart, Vivian J. Bardwell, Ricky W. Johnstone, Stephin J. Vervoort, Lev M. Kats

**Affiliations:** 1 The Peter MacCallum Cancer Centre, Melbourne, VIC 3000 Australia; 2 The Sir Peter MacCallum Department of Oncology, University of Melbourne, Parkville, VIC 3052 Australia; 3 Monash Haematology, Monash Health and School of Clinical Sciences at Monash Health, Monash University, Clayton, VIC 3168 Australia; 4 Department of Genetics, Cell Biology and Development and the Masonic Cancer Center, University of Minnesota, Minneapolis, MN 55455 USA

## Abstract

The BCL6 Corepressor (BCOR) is a component of a variant Polycomb repressive complex 1 (PRC1) that is essential for normal development. Recurrent mutations in the *BCOR* gene have been identified in acute myeloid leukaemia and myelodysplastic syndrome among other cancers; however, its function remains poorly understood. Here we examine the role of BCOR in haematopoiesis in vivo using a conditional mouse model that mimics the mutations observed in haematological malignancies. Inactivation of *Bcor* in haematopoietic stem cells (HSCs) results in expansion of myeloid progenitors and co-operates with oncogenic *Kras*^*G12D*^ in the initiation of an aggressive and fully transplantable acute leukaemia. Gene expression analysis and chromatin immunoprecipitation sequencing reveals differential regulation of a subset of PRC1-target genes including HSC-associated transcription factors such as *Hoxa7/9*. This study provides mechanistic understanding of how BCOR regulates cell fate decisions and how loss of function contributes to the development of leukaemia.

## Abstract (web summary)

BCL6 corepressor (BCOR) is recurrently mutated in acute myeloid leukaemia and myelodysplastic syndrome. Here, the authors use mouse models to show the mechanism of how inactivation of BCOR in haematopoietic stem cells contributes to the development of leukaemia.

## Introduction

The *BCL6 Co-repressor* (*BCOR*) gene is located on chromosome X and encodes a ~180 kDa nuclear protein that is ubiquitously expressed across adult human tissues. BCOR is essential for normal embryonic development; germline hemizygous *BCOR* loss-of-function mutations are responsible for male lethality while heterozygous mutations in females result in oculocardiofaciodental (OFCD) syndrome, a rare genetic condition characterised by craniofacial, ocular and cardiac abnormalities^1^. Next-generation sequencing studies have demonstrated that somatic *BCOR* mutations occur in a range of conditions that affect the myeloid and erythroid haematopoietic lineages including acute myeloid leukaemia (AML), myelodysplastic syndrome (MDS), chronic myelomonocytic leukaemia (CMML) and aplastic anaemia^2–6^. The mutations in these diseases almost always result in a premature stop codon and nonsense-mediated decay or protein truncation, strongly suggestive of a tumour suppressor role for BCOR in these contexts. Interestingly however, BCOR can mediate pro-oncogenic functions in some cell types, while in other contexts it behaves as a tumour suppressor^7–11^. Hence, the role of *BCOR* is highly tissue-specific and should be functionally analysed in a physiologically relevant setting that is appropriate for the disease being studied.

Polycomb group (PcG) proteins are evolutionary conserved chromatin modifiers that regulate a broad array of genes in mammals and play major roles in development and cancer^12^. PcG proteins are present in multi-protein complexes that can be classified into two types, Polycomb repressive complex 1 and −2 (PRC1 and −2). PRC1 and PRC2 possess distinct enzymatic activities: PRC1 ubiquitinates histone 2A at lysine 119 (H2AK119ub) whereas PRC2 di- and trimethylates lysine 27 of histone 3 (H3K27me2/3). The canonical pathway of PRC transcriptional repression, established predominantly from studies in *Drosophila* and mammalian embryonic stem cells, involves the sequential recruitment and activity of PRC2 followed by PRC1^12,13^. However, PRC1 complex components are known to be highly variable and at least six distinct complexes have been described. Each complex is composed of a catalytic ubiquitin-ligase core containing RING1/RNF2 (also known as RING1A/B) and a Polycomb Group Ring Finger (PCGF) paralogue, bound to different accessory proteins^12,13^. The mode of recruitment of these distinct entities to chromatin, their effects on the regulation of gene expression and their distinct biological functions remain under investigation.

The BCOR protein was initially identified in a yeast two-hybrid screen as an interaction partner of the transcription factor (TF) B-cell Lymphoma 6 (BCL-6)^14^ and was subsequently shown to be a member of a non-canonical PRC1 complex. In multiple different cell types BCOR co-purifies with RING1/RNF2, PCGF1, RYBP, SKP1 and the histone demethylase KDM2B, a complex commonly referred to as PRC1.1^15–18^. Interestingly, in germinal centre B cells, BCOR can assemble into an alternate CBX8 containing Polycomb complex^7^ underscoring the context-dependent nature of PRC1 complexes. The C-terminal PCGF Ub-like fold discriminator (PUFD) domain of BCOR is necessary and sufficient for its interaction with PRC1 and can bind to PCGF1 or −3^19^. BCOR has also been shown to directly bind AF9, although whether this occurs outside of a PRC1 context is unknown^20^.

Herein, a conditional mouse model of *Bcor* inactivation was developed to explore its function in myeloid differentiation and transformation. Using small numbers of haematopoietic cells isolated ex vivo, comprehensive analyses of the transcriptional and epigenetic consequences of *Bcor* loss were conducted. We demonstrate that *Bcor* has a pivotal role in the regulation of haematopoietic stem cell (HSC) associated transcriptional networks. Loss of Bcor results in expansion of myeloid progenitor cells, and in the context of oncogenic *Kras*, the initiation of cancer.

## Results

### Bcor mutation leads to expansion of myeloid progenitors

**Fig. 1:**
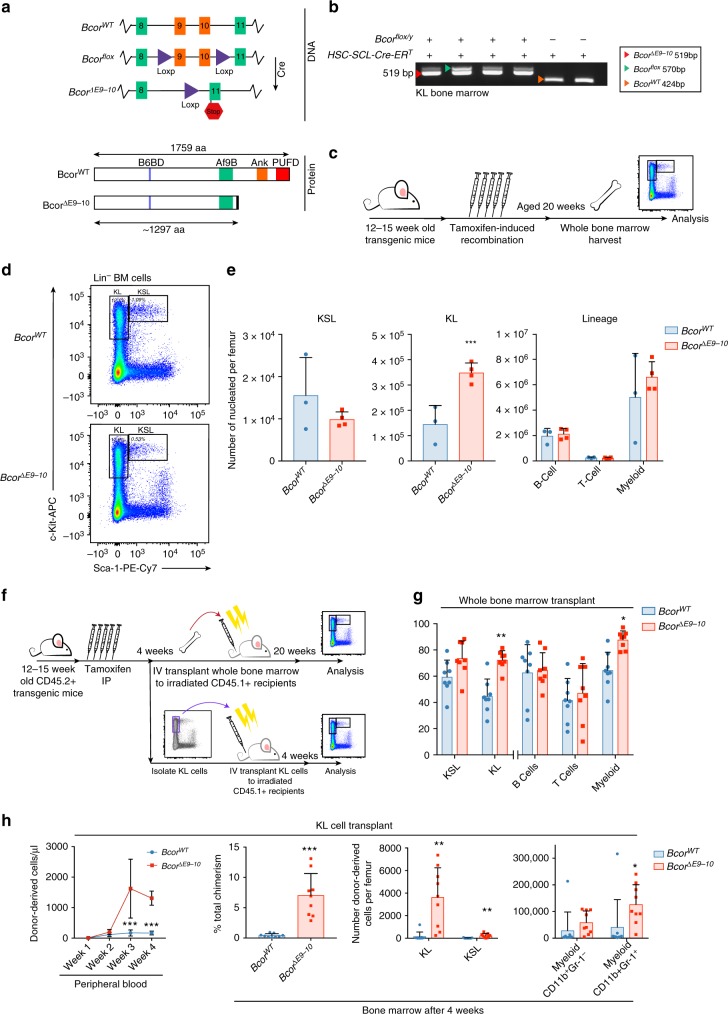
*Bcor* mutation increases repopulating capacity of myeloid progenitors. **a** Diagram of *Bcor* wild-type and conditional alleles (middle, exons 9–11 labelled) and the encoded truncated protein resulting from Cre-mediated recombination (bottom, protein domains labelled; B6BD, BCL-6 binding domain; Af9B, Af9 binding domain (present only in isoforms a and b) Ank Ankyrin repeat, PUFD PCGF ub-like fold discriminator, black box denotes 11 out-of-frame amino acids before stop codon). **b** PCR genotyping of sorted KL (Kit^+^Sca1^−^Lin^−^) cells performed 20 weeks post tamoxifen administration. PCR products corresponding to *Bcor*^*ΔE9-10*^ (red arrow), *Bcor*^*flox*^ (green arrow), and *Bcor*^*WT*^ (orange arrow) are shown. **c** Schematic overview of analysis of *Bcor* ^*ΔE9-10*^ and *Bcor*^*WT*^ mice. **d** Representative flow cytometry plot of bone marrow (BM) from *Bcor*^*ΔE9-10*^ and *Bcor*^*WT*^ mice at 20 weeks. The strategy for gating KL (Kit^+^Sca1^−^Lin^−^) and KSL (Kit^+^Sca1^+^Lin^−^) cells within lineage negative (CD5^−^, B220^−^, CD11b^−^, Gr-1^−^, 7–4^−^ and Ter-119^−^) BM cells is indicated. **e** Absolute numbers of indicated cell populations per femur quantified by flow cytometry (*n* = 3–4 mice/genotype; KL *p* = 0.0049, KSL *p* = not significant (N.S.)). **f** Schematic overview of transplantation experiments using total BM mononuclear cells or isolated BM KL cells. **g** Percentage of donor-derived cells (CD45.2^+^) within the indicated populations in the BM of *Ptprca* recipients transplanted with total BM mononuclear cells quantified by flow cytometry 20 weeks post-transplant (*n* = 8 recipients, KL: *p* = 0.00012; myeloid: *p* = 0.0075). **h** Absolute numbers and percentage chimerism of donor-derived cells in the peripheral blood and BM of *Ptprca* recipients transplanted with sorted KL cells quantified by flow cytometry (*n* = 9 recipients; PB week3, *p* = 2.084E-12, week 4, *p* = 3.84E-09, BM chimerism *p* = 4.0E-4, KL *p* = 1.2E-3, KSL *p* = 3.7E-3, Myeloid CD11b^+^only *p* = Myeloid CD11b^+^Gr1^+^ *p* = 2.3E-2). Values from individual animals are shown, bars indicate mean ±standard deviation, means compared with unpaired *t*-test: *p < 0.05, ** *p* <  0.005, *** *p* < 0.0005)

To analyse the function of *Bcor* in haematopoiesis in vivo a novel conditional mouse model was generated that mimics the truncating *BCOR* mutations observed in AML. We made use of a previously developed *Bcor*^*flox*^ allele that has exons 9 and 10 flanked by *LoxP* sites (Fig. 1a) (Hamline et al., in prep.). Cre recombinase-mediated excision of exons 9 and 10 causes a frameshift and the introduction of a premature in-frame stop codon in the *Bcor* open reading frame. The recombined *Bcor*^*flox*^ locus encodes a truncated Bcor protein that lacks the PUFD domain required for interaction with the PRC1 complex^19^ (Fig. 1a).

*Bcor*^*flox*^ mice were crossed with *HSC-SCL-Cre-ER*^*T*^ mice to facilitate tamoxifen inducible *Bcor* deletion specifically in HSCs^21^. Administration of tamoxifen to a cohort of *Bcor*^*flox*^;*HSC-SCL-Cre-ER*^*T*^ animals resulted in efficient recombination at the *Bcor* locus (Fig. 1b, Supplementary Fig. 1A-B) (hereafter referred to as *Bcor*^*ΔE9-10*^). *Bcor*^*ΔE9-10*^ mice were compared to tamoxifen treated single transgenic *Bcor*^*flox*^ or *HSC-SCL-Cre-ER*^*T*^ littermates which expressed wild-type Bcor (hereafter referred to as *Bcor*^*WT*^). Using immunobotting we confirmed that *Bcor*^*ΔE9-10*^ results in low level expression of a truncated protein (Supplementary Fig. 1C), consistent with previously published findings of the same allele^8^.

*Bcor*^*ΔE9-10*^ mice did not display overt haematopoietic dysfunction over a twenty-week observation period and their peripheral blood counts remained within normal limits (Supplementary Fig. 1D). To identify more subtle phenotypes in *Bcor*^*ΔE9-10*^ mice, a comprehensive immuno-phenotypic analysis of bone marrow populations was performed (Fig. 1c). There was a significant increase in the absolute number of cKit^+^Sca1^−^Lin^−^ myeloid progenitor cells (KL cells) in the bone marrow *Bcor*^*ΔE9-10*^ mice, whilst there was no difference in the number of cells in the cKit^+^Sca1^+^Lin^−^ HSC compartment (KSL cells) (Fig. 1d, e and Supplementary Fig. 1E, J). Further analysis of the sub-populations that make up the KSL compartment using a previously published gating strategy^22^ (Supplementary Fig. 1F) demonstrated no significant difference in long term haematopoietic stem cells (LT-HSC), multi potent progenitor cells (MPP) or haematopoietic progenitor cells (HPC1/HPC2) (Supplementary Fig. 1G). The absence of an effect in KSL cells was not due to inefficient gene knockout, as comparable recombination of the *Bcor* locus was observed in both KL and KSL cells at 12 weeks (Supplementary Fig. 1A, B) post tamoxifen administration.

To further investigate the effect of *Bcor* mutation on haematopoiesis, competitive transplantation experiments were performed in congenic *Ptprca* mice (Fig. 1f). First, bone marrow cells from tamoxifen treated *Bcor*^*ΔE9-10*^ or *Bcor*^*WT*^ donor mice were transplanted into sub-lethally irradiated *Ptprca* recipients and the level of leukocyte chimerism in the bone marrow was assessed after 20 weeks. *Bcor*^*ΔE9-10*^ cells were capable of tri-lineage differentiation, but also demonstrated an expansion of KL cells and an increase in mature myeloid cells compared with *Bcor*^*WT*^ cells (Fig.1g, Supplementary Fig. 1H). As there was no difference between *Bcor*^*ΔE9-10*^ and *Bcor*^*WT*^ KSL cells either at steady state or in the transplant setting, we hypothesised that the increased KL compartment in *Bcor*^*ΔE9-10*^ animals was due to an enhanced self-renewal or proliferative capacity of *Bcor*^*ΔE9-10*^ KL cells. To evaluate this, KL cells were isolated from *Bcor*^*ΔE9-10*^ *or Bcor*^*WT*^ donor mice and transplanted into sub-lethally irradiated *Ptprca* recipients (Fig. 1f). The absolute number of donor-derived cells in the peripheral blood was tracked over 4 weeks after which point the bone marrow was assessed for donor-derived populations. *Bcor*^*ΔE9-10*^ KL cells displayed enhanced repopulating capacity, expanding to more than three times the size of the *Bcor*^*WT*^ population in the peripheral blood of recipients over the 4-week period (Fig. 1h, Supplementary Fig. 1I). Further investigation revealed that the transplanted *Bcor*^*ΔE9-10*^ KL cells remained in the bone marrow after 4 weeks, whereas *Bcor*^*WT*^ cells were largely depleted (Fig. 1h). Additionally, recipients transplanted with *Bcor*^*ΔE9-10*^ KL cells had higher numbers of donor-derived mature myeloid cells compared to those recipients that received *Bcor*^*WT*^ KL cells (Fig. 1h, Supplementary Fig. 1I). Taken together, these data demonstrate that *Bcor* mutation in HSCs results in increased self-renewal and an expansion of myeloid progenitor cells in vivo.

### Bcor regulates an HSC-associated transcriptional program

**Fig. 2:**
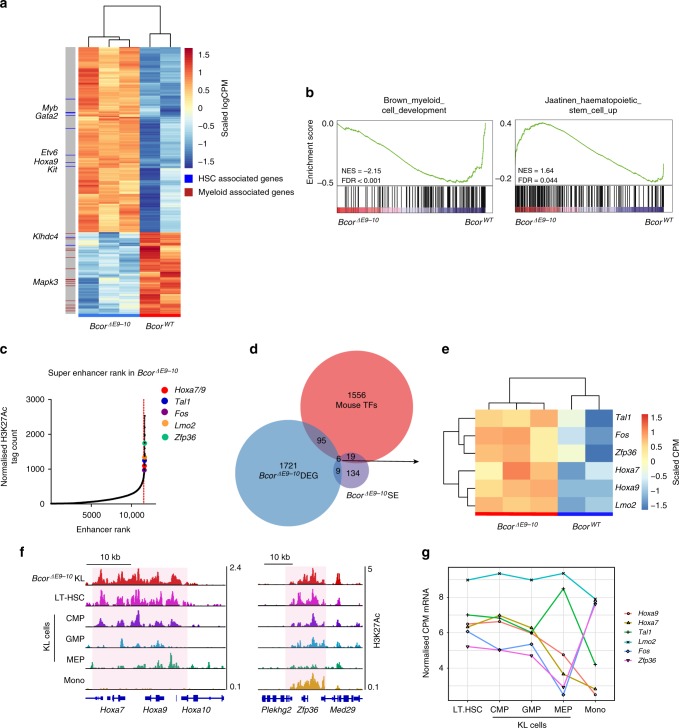
Bcor regulates master HSC TFs. **a**, **b** Quant-seq was performed on KL cells sorted from the BM of *Bcor*^*ΔE9-10*^ and *Bcor*^*WT*^ mice at 20 weeks post tamoxifen administration. **a** Heatmap of normalized log counts per million (CPM) of the 500 most significantly differentially expressed genes. Genes associated with myeloid or HSC GSEA gene sets are indicated. **b** GSEA enrichment plots comparing Quant-seq from *Bcor*^*ΔE9-10*^ and *Bcor*^*WT*^ KL cells against myeloid cell development and HSC gene sets. FDR False discovery rate, NES Normalized enrichment score. **c**–**e** Super-enhancer analysis based on H3K27ac ChIP-seq data in *Bcor*^*ΔE9-10*^ KL cells. **c** All enhancers identified in one representative *Bcor*^*ΔE9-10*^ sample ranked by H3K27ac tag count. Red dashed line indicates a super-enhancer slope of 1. Genes associated with super-enhancers identified in 2/3 *Bcor*^*ΔE9-10*^ samples and differentially expressed in *Bcor*^*ΔE9-10*^ KL cells compared with *Bcor*^*WT*^ KL cells have been highlighted. **d** Venn diagram showing overlap of genes significantly differentially expressed in *Bcor*^*ΔE9-10*^ compared with *Bcor*^*WT*^ KL cells (*p* < 0.05, absolute logFC >0.5), genes associated with SEs identified in 2/3 *Bcor*^*ΔE9-10*^ samples, and mouse transcription factors from RIKEN transcription factor database^68^ (DEG Differentially expressed genes, TFs transcription factors, SE super-enhancer). **e** Heatmap of row-scaled Quant-seq counts for six differentially expressed, super-enhancer-associated genes encoding transcription factors. **f** H3K27ac ChIP-seq read density for the *Hoxa7*, *Hoxa9* and *Zfp36* loci in the indicated haematopoietic cell populations^25^. **g** Line graph showing normalized RNA expression for super-enhancer-associated *Bcor*-regulated transcription factor genes across in the indicated haematopoietic cell populations^25^

In order to discern the molecular mechanisms that underpin the aberrant phenotype of *Bcor*^*ΔE9-10*^ myeloid progenitors, gene expression analysis using 3′ RNA sequencing (Quant-seq) was performed on KL cells isolated from *Bcor*^*ΔE9-10*^ and *Bcor*^*WT*^ mice. We identified 1497 significantly differentially expressed genes (DEG; see Supplementary Data 1) including many genes involved in regulation of haematopoiesis, leukocyte differentiation and activation (Fig. 2a and Supplementary Fig. 2A and B). Gene set enrichment analysis (GSEA) revealed that *Bcor*^*ΔE9-10*^ KL cells expressed higher levels of transcripts that are typically enriched in HSCs, and conversely expressed lower levels of transcripts that are upregulated during normal myeloid development (Fig. 2a, b). Quant-seq was also performed on donor-derived *Bcor*^*ΔE9-10*^ and *Bcor*^*WT*^ KL cells isolated from *Ptprca* recipient mice. Comparison of the two independently derived data sets demonstrated a high degree of correlation between transcriptional changes in the transplant and primary transgenic settings (Supplementary Fig. 2C). These data suggest that Bcor is partially responsible for reprogramming the transcriptome during myeloid commitment and that *Bcor* mutation results in aberrant expression of a stem cell-like transcriptional program in myeloid progenitor cells.

To identify genes that may mediate these effects, we focused on differentially expressed TFs, as these proteins play a key role in controlling cell-type-specific gene expression. In total 89 TFs were differentially expressed between *Bcor*^*ΔE9-10*^ and *Bcor*^*WT*^ KL cells (Fig. 2b and Supplementary Table 4). These included *Runx2*, *Myc* and multiple members of the *Gata*, *Cebp* and *Hoxa* families with established roles in myeloid differentiation.

Recent studies have demonstrated that an interconnected network of TFs the expression of which is regulated by super-enhancers (SEs) plays a key role in the establishment of cell identify^23,24^. We reasoned that defining the SE network in *Bcor*^*ΔE9-10*^ KL cells would aid in the identification of key downstream mediators of Bcor function. To that end we generated a ChIP-seq dataset for H3K27ac, a mark associated with active chromatin that is widely used to determine the location of enhancers and SEs^24^. A total of 168 SEs were identified in at least two out of the three *Bcor*^*ΔE9-10*^ samples analysed (Fig. 2c, Supplementary 2E). In agreement with the existing literature^24^, SE-associated genes were highly expressed (Supplementary Fig. 2D) and enriched for master haematopoietic TFs such as *Tal1* and *Fos*. We identified six SE-associated TFs that were differentially expressed and all were upregulated in *Bcor*^*ΔE9-10*^ KL cells compared with *Bcor*^*WT*^ KL cells (Fig. 2d, e). Analysis of publicly available H3K27ac ChIP-Seq and expression data from isolated mouse haematopoietic cell populations^25^ demonstrated that each of the six genes were also associated with both SEs and high expression in HSCs. Moreover, their expression and SE-status was lost during differentiation (Fig. 2f, g and Supplementary Fig. 2E), suggesting that they are key drivers of HSC identity and that their downregulation is important for lineage commitment.

### H2AK119ub is regulated during myeloid differentiation

**Fig. 3:**
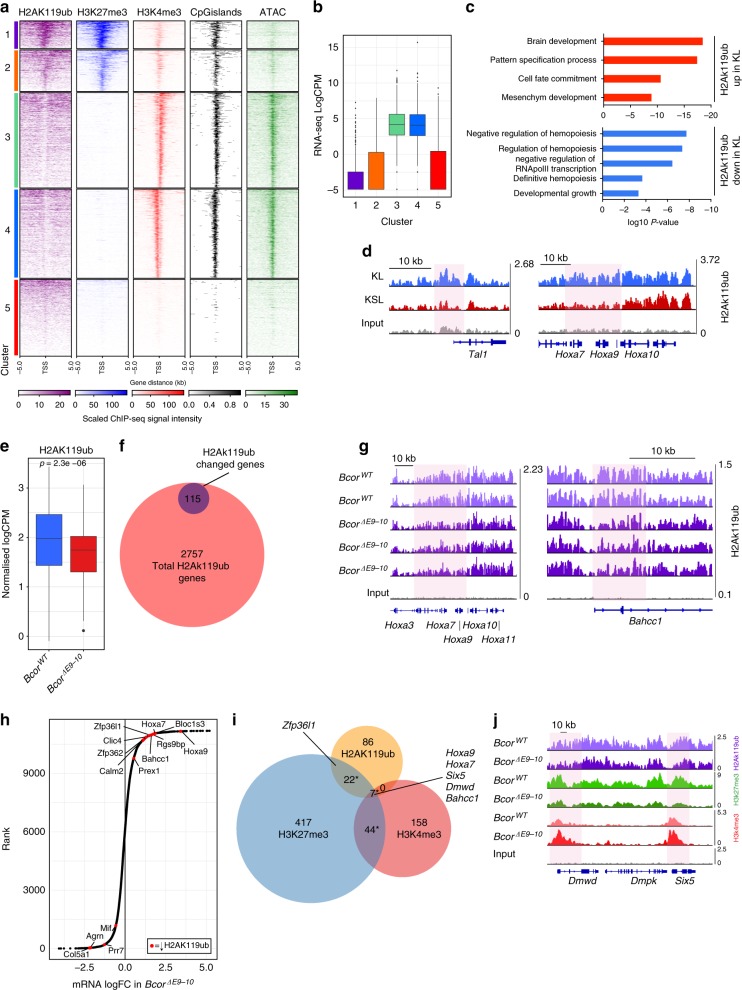
Bcor regulates H2AK119ub at a subset of PRC1 target genes. **a** Heatmap representing H2AK119ub, H3K27me3 and H3K4me3 occupancy, GpG island density (CpGi) and DNA accessibility (ATAC) at all annotated gene promoters (5 kb flanking TSSs of Refseq genes regardless of direction; scales indicated) in *Bcor*^*WT*^ KL cells, separated into 5 groups by K-means clustering. **b** Tukey boxplot of RNA expression in *Bcor*^*WT*^ KL cells for each of the 5 clusters identified in **a**. **c** Gene ontology analysis of genes associated with differentially ubiquitinated regions in *Bcor*^*WT*^ KL cells compared with *Bcor*^*WT*^ KSL cells (up in KL, log_2_FC >0.5; down in KL, log_2_FC <−0.5). **d** H2AK119ub ChIP-seq read density at *Tal1* and *Hoxa9* loci in *Bcor*^*WT*^ KL and KSL cells. Differentially ubiquitinated regions are highlighted in red. **e** Tukey boxplot of normalized H2AK119ub ChIP-seq signal from top 100 differentially ubiquitinated regions in *Bcor*^*ΔE9-10*^ and *Bcor*^*WT*^ KL cells (means compared with unpaired *T*-test, *p* = 2.03E-6.) **f** Venn diagram overlaying all genes associated with H2AK119ub-enriched regions in KL cells and genes associated with differentially ubiquitinated regions in *Bcor*^*ΔE9-10*^ KL cells compared with *Bcor*^*WT*^ KL cells. **g** *Hoxa9* and *Bahcc1* gene loci showing H2AK119ub ChIP-seq read density in *Bcor*^*WT*^ and *Bcor*^*ΔE9-10*^ KL cells. Differentially ubiquitinated region is highlighted in red. **h** Ranked plot comparing RNA expression between *Bcor*^*ΔE9-10*^ and *Bcor*^*WT*^ KL cells. Genes that are significantly differentially expressed (log_2_FC >0.5, *p* < 0.05) and associated with decreased H2AK119ub (log_2_FC <−0.3, *p* < 0.01) in *Bcor*^*ΔE9-10*^ KL cells compared with *Bcor*^*WT*^ KL cells are highlighted. **i** Venn diagram showing genes associated with H3K27me3, H2AK119ub or H3K4me3 differentially enriched regions (logFC >0.3 or <−0.3, *p* < 0.05) in *Bcor*^*ΔE9-10*^ KL cells compared with *Bcor*^*WT*^ KL cells. Overlaps were assessed for significance by hypergeometric test^69^ (H3K27me3 and H2AK119ub *p* = 3.4E-22, H3K27me3 and H3K4me3 *p* = 1.8E-37, H2AK119ub and H3K4me3 *p* = 0.00015, see Supplementary Table 5). **j** *Dmwd* and *Six5* gene loci showing H2AK119ub, H3K27me3 and H3K4me3 ChIP-seq read density in *Bcor*^*ΔE9-10*^ and *Bcor*^*WT*^ KL cells. One representative sample per genotype is shown. For Tukey boxplots centre line denotes median, whiskers extend to 1.5xIQR

Bcor is a component of PRC1.1^15^ and inactivation of Bcor has been shown to impact on the activity of PRC1 and PRC2 at key loci^8,17^. This led us to initially characterise the genome-wide distribution of the PRC1-catalysed histone modification H2AK119ub and the PRC2-associated mark H3K27me3 in wild-type KL cells (Fig. 3a). To understand how these marks relate to other chromatin modifications and to chromatin accessibility, ChIP-seq for H3K4me3 (a modification associated with active transcription start sites) and Assay for Transposase-Accessible Chromatin using sequencing (ATAC-seq) were also performed. Analysis of the genomic distribution of H2AK119ub-enriched regions using a window-based method optimised for broad histone marks^26^ demonstrated enrichment around promoter proximal regions of genes, with a lesser degree of occupancy at genomic loci distal to transcribed genomic regions (Supplementary Fig. 3A, D). Therefore, subsequent analysis was focussed on regions surrounding transcription start sites (TSS). K-means clustering was used to define five clusters based on ChIP and ATAC-seq results (Fig. 3a) and the transcriptional state of each of these clusters was assessed (Fig. 3b). H2AK119ub and H3K27me3 signal was highly enriched in clusters 1 and 2 and genomic loci enriched for these two marks were significantly overlapping when assessed by permutation test (*p* < 0.001; see Supplementary Table 5). These clusters also had low H3K4me3 signal and low chromatin accessibility as measured by ATAC-seq indicating a ‘closed’ conformation. In contrast clusters 3 and 4 had high levels of H3K4me3, ‘open’ chromatin and low levels of H2AK119ub and H3K27me3. While PRC1.1 has been associated with CpGs^27^, no significant enrichment of CpG islands in H2AK119ub-containing clusters 1 or 2 compared to other clusters was identified. Furthermore, we found no evidence of H2AK119ub signal independent of H3K27me3 as has been previously suggested to be characteristic of PRC1.1 mediated H2AK119ub^10^, highlighting that in these cells H2AK119ub appears to be predominantly co-occurring with H3K27me3. Gene expression analysis confirmed that genes in clusters 3 and 4 were highly expressed, whereas those in clusters 1 and 2 had lower expression (Fig. 3b), consistent with the active and repressive nature of their promoters, respectively.

To investigate whether deposition of the H2AK119ub mark is dynamically regulated during differentiation we compared ChIP-seq data derived from wild-type KSL and KL cells. Analysis of H2AK119ub ChIP-seq data identified 2118 H2AK119 ubiquitinated regions that were significantly altered during differentiation from KSL to KL cells. Association of the differentially ubiquitinated regions with the nearest gene identified 561 genes that were enriched for differentiation-related gene ontology (GO) terms such as development, cell fate commitment and regulation of haemopoiesis (Fig. 3c). Importantly, we observed a gain of H2AK119ub signal in KL cells at key TF loci *Tal1*, *Hoxa7* and *Hoxa9* (Fig. 3d). Taken together these results demonstrate that H2AK119ub is associated with gene repression and is redistributed during HSC differentiation including at multiple cell identity-defining TFs.

### Bcor regulates H2AK119ub at a subset of PRC1 target genes

To understand how loss of Bcor impacts histone ubiquitination, the H2AK119ub distribution in *Bcor*^*ΔE9-10*^ and *Bcor*^*WT*^ KL cells was compared. Analysis identified 9216 regions enriched for H2AK119ub 3-fold above input threshold in *Bcor*^*WT*^ KL cells. Consistent with the hypothesis that PRC1.1 accounts for a fraction of overall PRC1 activity, we observed locus-specific changes rather than a global loss of H2AK119ub in *Bcor*^*ΔE9-10*^ KL cells. Analysis revealed 661 regions were significantly differentially ubiquitinated, predominantly within promoter regions of genes (Supplementary Fig. 3A). Notably, for the top 100 most differentially ubiquitinated loci, the majority were decreased in *Bcor*^*ΔE9-10*^ cells compared with controls (Fig. 3e and Supplementary Fig. 3B).

Next, we assigned each of the gene-proximal ubiquitinated regions in KL cells to the nearest gene. This analysis identified a total of 2757 H2AK119ub-associated genes, of which 115 were significantly altered in *Bcor*^*ΔE9-10*^ cells (Fig. 3f, Supplementary Data 2), including *Hoxa7*, *Hoxa9* and *Bahcc1* (Fig. 3g). Despite the small number of genes associated with differential ubiquitination, GO analysis demonstrated that they were highly enriched for hematopoietic processes including blood cell differentiation and cell fate commitment (Supplementary Fig. 3C). Loss of H2AK119ub was significantly associated with increased gene expression as assessed by hypergeometric testing (*p* =  0.012, Supplementary Table 5) and we identified 10 putative direct targets of PRC1.1., where loss of H2AK119ub was accompanied by upregulation of transcription (Fig. 3h). Of these identified target genes, six have known roles in transcriptional regulation, signalling or proliferation in cancer (*Hoxa9*^28^*, Hoxa7*^29^*, Prex1*^30^*, Clic4*^31^*, Zfp36l1*^32^ and *Calm2*^33^), and are therefore potentially contributing to the transcriptional reprogramming and expansion of *Bcor*^*ΔE9-10*^ myeloid progenitor cells.

H2AK119ub deposited by non-canonical PRC1 complexes has previously been shown to drive recruitment and activity of PRC2^34^. In order to investigate whether *Bcor* mutation was associated with alterations in H3K27me3 or H3K4me3, further ChIP-seq in *Bcor*^*ΔE9-10*^ and *Bcor*^*WT*^ KL cells was performed. Our analysis revealed no global change in H3K27me3 or H3K4me3 signal (Supplementary Fig. 3D), although as expected we did identify locus-specific differences (Fig. 3i). Interestingly, we observed a significant decrease in H3K27me3 signal in the top 100 differentially methylated regions, but this was not the case for regions where H2AK119ub was decreased in *Bcor*^*ΔE9-10*^ cells (Supplementary Fig. 3E). There was however, a significant overlap between genes associated with H2AK119ub changes and those associated with a change in H3K27me3 alterations as assessed by hypergeometric test (*p* = 3.4E-22, Fig. 3i, Supplementary Table 5). The changes in H3K4me3 were balanced (i.e. increased and decreased in equal measures) and there were no changes in signal either in the top 100 differentially methylated regions or in those regions where H2AK119ub was decreased in *Bcor*^*ΔE9-10*^ cells (Supplementary Fig. 3E). We did identify seven loci, including *Hoxa7*, *Hoxa9*, *Dmwd* and *Six5*, associated with alterations in all three marks (centre, Fig. 3i). These genes uniformly exhibited significant loss of both H2AK119ub and H3K27me3, and a gain of H3K4me3 (Fig. 3j), indicative of epigenetic activation. Alterations in H3K27me3 and H3K4me3 signal were significantly correlated with changes in mRNA expression (Supplementary Fig. 3F), suggesting these epigenetic changes may be indicative of consequential transcriptional changes downstream of *Bcor*^*ΔE9-10*^.

### Bcor^ΔE9-10^ and Kras^G12D^ co-operate to initiate leukaemia

**Fig. 4:**
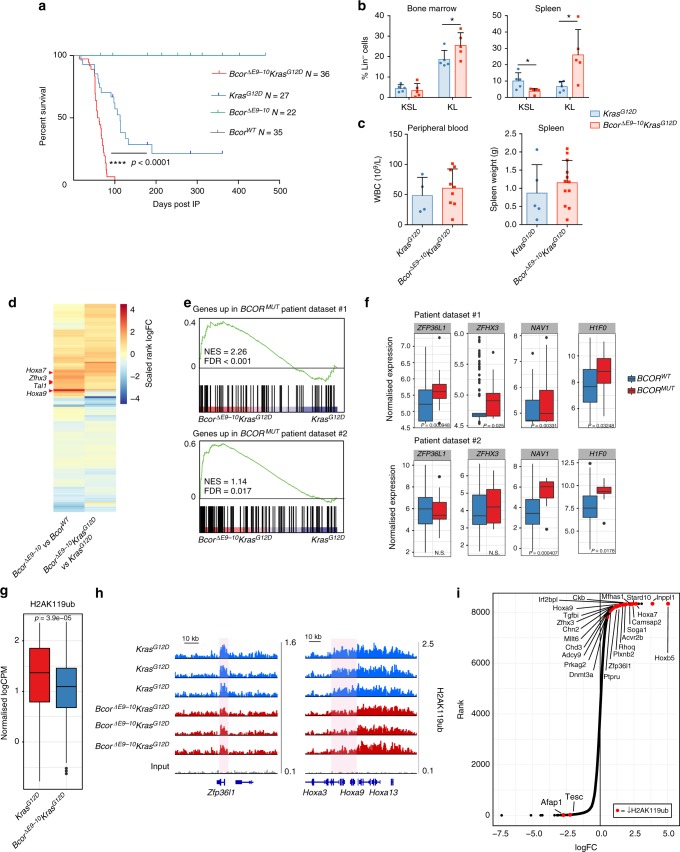
*Bcor*^*ΔE9-10*^ cooperates with *Kras*^*G12D*^ to initiate leukaemia. **a** Kaplan–Meier survival curve of primary transgenic mice following tamoxifen administration. (median survival for *Bcor*^*ΔE9-10*^*Kras*^*G12D*^ mice was 58 days compared with 113 days for *Kras*^*G12D*^ mice; survival compared with logrank Mantel–Cox test, *p* < 0.0001; median survival not reached for *Bcor*^*ΔE9-10*^ and *Bcor*^*WT*^ groups). **b** Percentage of KL and KSL populations in the bone marrow and spleen of moribund *Bcor*^*ΔE9-10*^*Kras*^*G12D*^ (*n* = 5) and *Kras*^*G12D*^ only (*n* =5) mice quantified by flow cytometry (means were compared using unpaired *t*-test BM KL *p* = 0.02, Spleen KSL *p* = 0.027 KL *p* = 0.024). **c** Peripheral white blood cell (WBC) counts and spleen weights of moribund transgenic mice (WBC: *Bcor*^*ΔE9-10*^*Kras*^*G12D*^ *n* = 9, *Kras*^*G12D*^ *n* = 4; spleen: *Bcor*^*ΔE9-10*^*Kras*^*G12D*^ *n* = 12, *Kras*^*G12D*^ *n* = 5). **d** Heatmap showing correlation in gene expression changes regulated by Bcor in the non-malignant and malignant contexts. The top 250 most significantly differentially expressed genes (comparing *Bcor*^*ΔE9-10*^*Kras*^*G12D*^ and *Kras*^*G12D*^ KL cells) are included. Correlation analysis was performed with Spearman’s rank correlation (*ρ* = 0.69, *p*-value <2.2E-16). **e** GSEA enrichment plots comparing Quant-seq from *Bcor*^*ΔE9-10*^*Kras*^*G12D*^ and *Kras*^*G12D*^ murine leukaemia against gene expression signatures derived from *BCOR*-mutant AML patients (see Methods). **f** Tukey boxplots of normalized expression of selected target genes in AML patient samples. **g** Tukey boxplot of normalized H2AK119ub ChIP-seq tag counts from the top 100 differentially ubiquitinated regions comparing *Bcor*^*ΔE9-10*^*Kras*^*G12D*^ and *Kras*^*G12D*^ KL cells (means compared with unpaired *t*-test, *p* = 3.9E-05). **h** *Zfp36l1* and *Hoxa9* gene loci showing H2AK119ub ChIP-seq read density in *Bcor*^*ΔE9-10*^*Kras*^*G12D*^ and *Kras*^*G12D*^ KL cells. Differentially ubiquitinated regions are highlighted in red. **i** Ranked plot comparing RNA expression between *Bcor*^*ΔE9-10*^*Kras*^*G12D*^ and *Kras*^*G12D*^ KL cells. Genes that are significantly differentially expressed (log_2_FC >0.5 or <−0.5, *p* < 0.05) and associated with decreased H2AK119ub (log_2_FC <−0.3, *p* < 0.05) in *Bcor*^*ΔE9-10*^*Kras*^*G12D*^ KL cells compared with *Kras*^*G12D*^ cells are highlighted. Values from individual animals are shown, bars indicate mean ± standard deviation. For Tukey boxplots centre line denotes median, whiskers extend to 1.5xIQR

To determine whether the phenotypic and molecular changes driven by *Bcor* inactivation can contribute to malignant transformation we aged a cohort of *Bcor*^*ΔE9-10*^ and control mice for over 12 months following tamoxifen injection. During this period, the animals did not develop leukaemia suggesting that inactivation of *Bcor* alone was not sufficient for cancer initiation (Fig. 4a). Mutations in epigenetic modifiers that alter haematopoietic differentiation have been shown to co-operate with dysregulated growth factor signalling to drive oncogenic transformation^22,35^ and *BCOR* mutations co-occur with RAS pathway mutations in both AML and MDS patients^2,36,37^. Thus, *Bcor*^*ΔE9-10*^ mice were crossed with *Lox-stop-lox-Kras*^*G12D*^ mice that express oncogenic *Kras* following excision of the transcriptional stop cassette by Cre (subsequently referred to as *Kras*^*G12D*^)^38^. After somatic recombination of both alleles in HSCs, *Kras*^*G12D*^ and *Bcor*^*ΔE9-10*^*Kras*^*G12D*^ mice developed a lethal haematopoietic disease characterised by leucocytosis, splenomegaly and increased leukaemic blasts in the peripheral blood and bone marrow (Fig. 4a, b, Supplementary Fig. 4A). Notably however, the survival of *Bcor*^*ΔE9-10*^ *Kras*^*G12D*^ mice was greatly reduced compared with *Kras*^*G12D*^ controls (Fig. 4a). Bone marrow cells from both moribund *Kras*^*G12D*^ and *Bcor*^*ΔE9-10*^*Kras*^*G12D*^ animals could recapitulate a lethal disease in primary and secondary recipients upon retransplantation (Supplementary Fig. 4B). Flow cytometry analysis revealed a significant expansion of the KL cell compartment in the bone marrow and spleen of *Bcor*^*ΔE9-10*^*Kras*^*G12D*^ compared to *Kras*^*G12D*^ animals (Fig. 4b), whilst mature lineage populations in both spleen and bone marrow were similar (Supplementary Fig. 4C). Our data formally demonstrates that *Bcor* is a tumour suppressor in the myeloid lineage and can co-operate with additional events to initiate and accelerate leukaemia in vivo.

### Bcor regulates H2AK119ub and gene expression in leukaemia

To establish whether *Bcor*^*ΔE9-10*^ regulates expression of the same genes in a leukaemic context expression of *Hoxa5*, *7* and *9* was assessed by qPCR in bone marrow from moribund animals. Upregulation of *Hoxa5*, *7* and *9* was confirmed in *Bcor*^*ΔE9-10*^*Kras*^*G12D*^ compound mice compared with *Kras*^*G12D*^ controls (Supplementary Fig. 4D) suggesting that the transcriptional consequences of *Bcor*^*ΔE9-10*^ may be conserved in the malignant setting.

To expand on these findings CD45.2^+^ bone marrow cells from donor *Bcor*^*ΔE9-10*^*Kras*^*G12D*^ and *Kras*^*G12D*^ mice that had comparable disease burden were transplanted into irradiated *Ptprca* recipients. Donor-derived KL cells were isolated and analysed by Quant-seq. Differential gene expression analysis identified 434 significantly altered genes between *Kras*^*G12D*^ and *Bcor*^*ΔE9-10*^*Kras*^*G12D*^ cells that were highly enriched for differentiation and development-associated GO terms (Supplementary Fig. 4F, see Supplementary Data 3). GSEA analysis revealed that a pattern specification gene set strongly associated with *Hox* genes was upregulated in *Bcor*^*ΔE9-10*^*Kras*^*G12D*^ cells (Supplementary Fig. 4E). Changes in gene expression following *Bcor* mutation in the non-transformed and malignant settings were significantly correlated (Fig. 4d, Supplementary Fig. 4G), with key TFs *Hoxa7*, *Hoxa9* and *Tal1* also upregulated in the context of leukaemia (Fig. 4d).

To assess the  relevance of the *Bcor*^*ΔE9-10*^*Kras*^*G12D*^ murine model to human AML, we utilised two previously published mRNA expression data sets^39–41^ and analysed differential expression between *BCOR*-mutant (*BCOR*^*MUT*^) and *BCOR* wild-type (*BCOR*^*WT*^) leukaemia patients. Patient data sets #1 and #2 contained 518 and 437 AML patient samples with 13 and 19 *BCOR*^*MUT*^ patients, respectively (*BCOR*^*MUT*^ frequency ~3%). GSEA analysis revealed that *BCOR*^*MUT*^ signatures derived independently from the two data sets were strongly enriched in *Bcor*^*ΔE9-10*^*Kras*^*G12D*^ cells compared with *Kras*^*G12D*^ cells (Fig. 4d). A number of relevant genes were identified as upregulated by *BCOR* mutation in both the mouse model and human patient data including *NAV1* and *H1F0* (Fig. 4e). *HOXA* family genes were not upregulated in *BCOR*^*MUT*^ patients, however, these genes are known to be upregulated by multiple independent mechanisms in AML^28,42^, thus introducing a confounding factor to our analysis. Overall, these results suggest that *Bcor*^*ΔE9-10*^ is a faithful model of *BCOR*^*MUT*^ AML.

We next assessed the distribution of H2AK119ub in leukaemic KL cells using ChIP-seq. We identified 9221 H2AK119ub-enriched regions, 1103 of which were differentially ubiquitinated between *Bcor*^*ΔE9-10*^*Kras*^*G12D*^ and *Kras*^*G12D*^ cells (See Supplementary Data 4). Consistent with our earlier findings, for the top 100 most differential regions there was a significant loss of H2KA119ub signal in *Bcor*^*ΔE9-10*^*Kras*^*G12D*^ cells (Fig. 4g). We assigned genes to H2AK119ub-enriched regions and identified 285 genes associated with differential ubiquitination (Fig. 4h, Supplementary Fig. 4H), including 17 genes associated with reduced ubiquitination in both the normal and leukaemic contexts (Supplementary Data 2 and 4). GO analysis of genes associated with differential ubiquitination revealed terms linked to embryonic morphogenesis, differentiation and proliferation (Supplementary Fig. 4I). H2AK119ub loss was significantly associated with increased gene expression when assessed by both hypergeometric testing (*p* = 9E-11) and GSEA (*p* = 0.0165; Supplementary Fig. 4J and Supplementary Table 5). We identified twenty-three putative PRC1.1 target genes that were associated with both a loss of H2AK119ub and upregulation of transcription in *Bcor*^*ΔE9-10*^*Kras*^*G12D*^ cells (Fig. 4i). Importantly, this included *Hoxa7*, *Hoxa9* and *Zfp36l1* that were also identified as putative PRC1.1 targets in the non-leukaemic context. Altogether, these data indicate that *Bcor*^*ΔE9-10*^-dependent loss of histone ubiquitination and transcriptional regulation is largely conserved in both normal and malignant haematopoiesis.

### Hoxa9 is essential for proliferation of Bcor^ΔE9-10^Kras^G12D^ cells

**Fig. 5:**
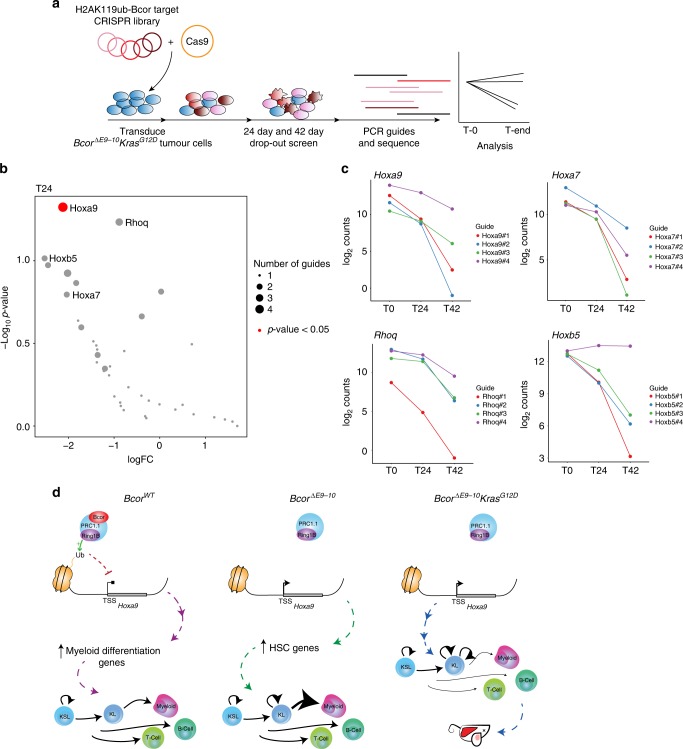
*Hoxa9* is essential for the proliferation of *Bcor*^*ΔE9-10*^*Kras*^*G12D*^ leukaemic cells. **a** Schematic overview of the CRISPR drop-out screen. **b** Negative enrichment of all genes in the CRISPR screen calculated by MAGeCK^65^ (negative enrichment for *Hoxa9* = 0.014268, *p* = 0.04737). **c** Selected sgRNA counts at T0, T24 and T42. **d** Model for Bcor function in haematopoietic differentiation and the initiation of leukaemia

To determine the impact of Bcor target genes on the proliferation of *Bcor*^*ΔE9-10*^*Kras*^*G12D*^ leukaemic cells a CRISPR drop-out screen was performed. We focussed on a subset of Bcor-regulated genes at which H2AK119ub loss was accompanied by increased transcription in *Bcor*^*ΔE9-10*^*Kras*^*G12D*^ cells. These genes were selected because they represent putative direct targets of PRC1.1. Primary *Bcor*^*ΔE9-10*^*Kras*^*G12D*^ tumour cells were extracted from the spleen of a moribund mouse and transduced with Cas9 followed by our custom sgRNA library (see Supplementary Data 5). Samples were collected post transduction and then after 24 and 42 days of in vitro culture, and guide representation determined by sequencing (Fig. 5a). Statistical analysis of sgRNAs that were significantly depleted at day 24 compared to day 0 revealed that guides targeting *Hoxa9* were significantly depleted (*p* = 0.04737, Fig. 5b, c, Supplementary Data 6). Whilst not statistically significant, the next highest scoring genes were *Rhoq, Hoxa7* and *Hoxb5* (Fig. 5b, c, Supplementary Data 6), indicating these genes may also be important for *Bcor*^*ΔE9-10*^*Kras*^*G12D*^ leukaemic cell survival. *Hox* family genes have been implicated in leukaemia previously^43^ and are therefore likely to be key drivers of *Bcor*^*ΔE9-10*^ phenotype. Guides targeting Dnmt3a were enriched at the end time point, indicating the upregulation of this gene is not essential to the *Bcor*^*ΔE9-10*^ phenotype and may even be antagonistic to some degree (Supplementary Fig. 5B, C). Accordingly, previous studies have demonstrated loss of *Dnmt3a* enhances self-renewal in HSCs^44^. Furthermore, these results were recapitulated when cells were harvested at 42 days (Supplementary Fig. 5D). Through these additional analyses, we can confirm that *Hoxa9* upregulation is essential for the leukaemic phenotype *of Bcor*^*ΔE9-10*^*Kras*^*G12D*^ tumours, and *Hoxa7* and *Hoxb5* may also be important.

## Discussion

*BCOR* is recurrently mutated in AML and a recent study implicated *Bcor* in the proliferation of myeloid cells in vitro^5^. The data presented here demonstrates an important role for Bcor in regulating myeloid differentiation and leukaemogenesis in vivo. Loss of Bcor function resulted in expansion of myeloid progenitor cells in *Bcor*^*ΔE9-10*^ mice and competitive transplantation experiments confirmed that *Bcor-*mutant myeloid progenitors outcompeted their wild-type counterparts. Notably, isolated *Bcor*^*ΔE9-10*^ KL cells exhibited enhanced repopulating capacity in vivo, suggesting that Bcor may function to restrict self-renewal in lineage committed myeloid progenitors.

Consistent with the observed phenotype, RNA sequencing revealed that ex vivo sorted *Bcor*^*ΔE9-10*^ KL cells were characterised by an HSC-like transcriptional signature. Loss of Bcor function was accompanied by broad gene expression changes and ~1500 Bcor-regulated genes were identified. Of particular note, we observed upregulation of six SE-associated TFs: *Hoxa7*, *Hoxa9, Lmo2*, *Fos*, *Tal1* and *Zfp36*. Each of these genes have well-established roles in both normal haematopoiesis and the initiation and maintenance of leukaemia^28,29,45–51^. However, we cannot exclude the contribution of other DEGs to the *Bcor*^*ΔE9-10*^ phenotype.

Bcor is a known component of the PRC1.1 complex^15,16,52^ and has been suggested to modulate H2AK119ub at key loci in myeloid cells^8^. This led us to examine the impact of Bcor loss on the distribution of H2AK119ub in KL cells. The catalytic components of PRC1, RING1 and RNF2, are essential for normal and leukaemic cells, implying that H2AK119ub is required for cell survival^53^. In contrast, altered expression of other PRC1 members, such as upregulation of BMI1 and CBX7 and downregulation of MEL18, are known to play a role in malignant progression^54–58^, suggesting that altered PRC1 function may promote leukaemogenesis. Consistent with the proposed role of BCOR as a member of the PRC1.1 complex^15,19^, *Bcor*^*ΔE9-10*^ KL cells had reduced levels of H2AK119ub at a small subset of ubiquitin-associated genes. Loss of H2AK119ub was significantly associated with increased transcription in malignant and non-transformed *Bcor*^*ΔE9-10*^ KL cells and we identified putative direct targets of PRC1.1 including *Hoxa7*, *Hoxa9* and *Zfp36l1*. The contribution of *Hoxa7* and *9* to cancer are well established^29,59^ and *Zfp36l1*, which encodes butyrate response factor 1, has been implicated in myeloid differentiation and negative regulation of the anti-apoptotic protein BCL2^32,60^. It is tempting to speculate that these genes are the initial drivers of transcriptional reprogramming in Bcor-mutant KL cells and indeed we found that *Hoxa9* is indispensable for the proliferation of *Bcor*^*ΔE9-10*^*Kras*^*G12D*^ leukaemic cells. However, Bcor-driven differential gene expression cannot solely be explained by PCR1.1 activity and we also identified key haematopoietic factors such as *Tal1*, *Lmo2* and *Fos* that were not associated with H2AK119ub changes. Altered expression of these genes in *Bcor*^*ΔE9-10*^ cells may be the result of Bcor transcriptional regulation that is independent of the ubiquitin-ligase activity of PRC1.1 or the downstream effects of primary Bcor targets.

The interaction between PRC1 and 2 and the requirement for catalytic activity to mediate transcriptional repression at target genes is not well defined. Consistent with previous studies^10^ we found a robust genome-wide association between H2AK119ub and H3K27me3 at proximal promoters of target genes suggesting that the two complexes predominantly act in tandem. There was a significant overlap between genes associated with changes in H2AK119ub and those with changes in H3K27me3. However, actual genomic regions at which H2A ubiquitination was altered in *Bcor*^*ΔE9-10*^ cells, did not exhibit a significant difference in H3K27 methylation. Interestingly, there were also many loci where H3K27me3 loss was not accompanied by a reduction in H2AK119ub signal. Whether this is directly or indirectly mediated by Bcor is unclear, but one intriguing possibility is that the PRC1.1 complex has an H2AK119ub-independent impact on PRC2 recruitment or activity^17^.

Functional studies across multiple systems suggest that BCOR is capable of both oncogenic and tumour suppressive functions, even in closely related cell types^7–10,61^. Here we generated a clinically-relevant model and showed that Bcor inactivation resulted in functional co-operation with oncogenic Kras to initiate leukaemia in vivo. Interestingly, a recent study that used an internal deletion mutant of *Bcor* found that removal of the BCL6-binding domain also has an impact on haematopoietic differentiation and the development of T-cell leukaemia^61^. While specific deletion or inactivation of the BCL6-binding domain has not been described in human cancer to date, we cannot exclude the possibility that Bcor has additional important functions independent of histone ubiquitination or polycomb binding. As protein expression from the *Bcor*^*ΔE9-10*^ allele is severely reduced, these additional functions are likely to be abrogated in our model as well, and thus also contribute to the phenotype we observe.

In summary, our work identifies BCOR as an important regulator of normal and malignant haematopoiesis. BCOR controls the expression of key HSC TFs, in part through ubiquitin-mediated silencing during myeloid differentiation. Accordingly, loss of Bcor function results in enhanced self-renewal of myeloid progenitors and in the context of other oncogenes, promotes malignant progression.

## Methods

### Animals

The Peter MacCallum Cancer Centre Animal Ethics Committee approved all in vivo procedures in this study. Complete details of the generation of the *Bcor*^*flox*^ allele will be described elsewhere (Hamline et al. in prep). In short, *loxP* sites flanking exons 9 and 10 of the *Bcor* gene were inserted into the mouse genome. Cre-mediated excision of exons 9 and 10 results in a frameshift and the introduction of a premature stop codon in the *Bcor* open reading frame. *HSC-SCL-Cre-ER*^*T*^ mice (also known as Tg(Tal1-cre/ERT)42–056Jrg) and *Lox-stop-lox-Kras*^*G12D*^ mice (also known as *Kras*^*tm4Tyj*^) were obtained from Jackson Labs. To activate Cre recombinase 3–6-month-old mice were dosed with a total of 5 mg tamoxifen suspended in corn oil (1% tamoxifen, 10% ethanol, 89% corn oil) via intra-peritoneal (IP) injection over five consecutive days. Both male and female animals were used for immunophenotyping, whereas only male animals were used for molecular analysis (including Quant-seq, ChIP-seq and ATAC-seq). Congenic C57BL/6.SJL-Ptprca (referred to throughout the text as *Ptprca*, a congenic C57BL/6 strain that expresses the differential pan leukocyte marker CD45.1) were purchased from the Animal Resources Centre of Peter MacCallum Cancer Centre or the Walter and Eliza Hall Institute of Medical Research (Melbourne, Australia). All experimental mice were maintained under specific pathogen-free conditions.

### Genotyping

Mice were initially genotyped at three weeks of age and additional analyses performed at endpoin. Genomic DNA was extracted from ear clippings, tail clippings bone marrow or spleen cells using DNeasy Blood & Tissue Kit (Qiagen). Genotyping was performed with gene specific primers (Supplementary Table 3) and Phusion DNA polymerase (Thermo Fisher) according to manufacturer’s instructions. Recombination of the *Bcor* locus was quantified by qPCR using the SYBRgreen High-ROX mix (Agilent) on a LightCycler LC480 (Roche) with primers internal to the deletion site (*Bcor* exon 9); P primers targeting *Bcor* exon 6 were used for normalization (Supplementary Table 3).

### Flow cytometry analysis

Single-cell suspensions of whole blood, bone marrow and spleen were incubated in ACK red cell lysis buffer (150mM NH4Cl, 10mM KHCO3, 0.1mM EDTA) to lyse erythrocytes and isolate nucleated cells. Cells were resuspended in FACS buffer (2% foetal calf serum (FCS; [Thermo Fisher] in phosphate buffered saline[PBS]) and stained with fluorophore-conjugated antibodies targeted against cell surface markers (Supplementary Table 1) on ice for 15 min. Cells were then washed with FACS buffer and immediately analysed on BD LSR Fortessa X-20 or BD LSR II flow cytometers (BD).

### Isolation of myeloid progenitor cells

Bone marrow was harvested from male transgenic mice 20 weeks post tamoxifen administration. Bone marrow cells were incubated with ACK red cell lysis buffer to remove erythrocytes and depletion of lineage positive cells was performed using the MACS Lineage Cell Depletion kit for mouse (Miltenyi Biotech, Cat#130–090–858) and magnetic LS columns (Miltenyi Biotech, Cat#130–042–401) according to the manufacturer’s instructions. Lineage depleted cells were stained with fluorophore-conjugated antibodies targeted against cell surface markers (Supplementary Table 1) as above and 4′,6-Diamidino-2-Phenylindole (DAPI) was used as a viability marker. Viable KL (cKit^+^Sca1^−^Lin^−^) were sorted on BD FACSAria Fusion 5, BD FACSAria II and BD FACSAria Fusion 3 (BD Biosciences) and used for transplantation and molecular analyses.

### Cell culture and lentiviral transduction

Sorted KL cells or single-cell suspensions from the spleens of tumour bearing *Bcor*^*ΔE9-10*^Kras^*G12D*^ mice (referred to as BK cells) were cultured in Dulbecco's Modified Eagle Medium (DMEM) supplemented with penicillin/streptomycin, 20% FCS, L-Asparagine (0.1mM), IL-3 (2ng/ml), IL-6 (2ng/ml), and SCF (10ng/ml). Cells were maintained at 37 °C and 10% CO_2_. Cells were genotyped as above to confirm their identity and were tested for *Mycoplasma* contamination. For transduction of BK cells, lentiviral supernatants were harvested from packaging 293T cells transfected with the custom CRISPR library (see below). Lentiviral particles were centrifuged onto RetroNectin-coated (Takara) non-treated tissue-culture plates and virus infection performed according the manufacturer’s protocol.

### In vivo competitive transplantation assays

Female *Ptprca* recipients were sub-lethally irradiated (5.5Gy administered as a single dose) using an X-RAD 320 irradiator (Precision X-Ray Incorporated) and sorted KL cells (5 × 10^4^) or bone marrow mononuclear cells (5 × 10^5^) from donor animals were inoculated via tail vein injection. Chimerism in peripheral blood and bone marrow was assessed by flow cytometry using the markers CD45.1 (recipient) and CD45.2 (donor).

### Western blotting

Cells were lysed in Laemmli buffer containing β-mercaptoethanol, subjected to SDS–PAGE on 4–20% Mini-PROTEAN TGX precast polyacrylamide gels (Bio-Rad Cat#4561093) and transferred to PVDF membrane (Millipore). Membranes were incubated with primary Bcor or β-actin antibodies (see Supplementary Table 2 for details) followed by anti-rabbit or anti-mouse IgG HRP-conjugated antibodies (Dako) Blots were developed with enhanced chemo-luminescence (ECL) reagents (GE Healthcare Cat#RPN2106) and exposed to film.

### In vitro CRISPR screen

Target genes were identified by integrating Quant-seq and H2AK119ub ChIP-seq data using the following selection criteria: increased mRNA expression (log_2_FC >0.5 and *p* < 0.05) and decreased H2AK119ub in the proximal promoter (log_2_FC <−0.3 and *p* < 0.05) in *Bcor*^*ΔE9-10*^*Kras*^*G12D*^ cells compared with *Kras*^*G12D*^ cells. We designed a custom CRISPR library targeting 25 genes (4 guides per gene) with 10 non-targeting control guides (guide sequences are provided in Supplementary Table 5). Guide sequences were PCR amplified from a CustomArray Inc oligo pool and cloned into the lentiGuide-Crimson lentiviral vector (Addgene plasmid #70683) using Golden Gate cloning^62^. The representation of all guides in the pool was validated by sequencing on a MiSeq (Illumina) with single-end 50 bp sequencing. BK cells were transduced using FUCas9Cherry (Addgene plasmid #70182) and Cherry^+^ cells were sorted on a BD FACSAria Fusion 5 (BD Biosciences). 8 × 10^6^ BK^Cas9^ cells were transduced with a multiplicity of infection of <0.1 to achieve single sgRNA integration per cell at an average 500-fold representation. Five days post viral infection Crimson^+^ cells were sorted on a BD FACSAria Fusion 5 (BD Biosciences) and returned to culture. Samples were then collected at four days (T0), 28 days (T24) and 46 days (T42) post sorting and genomic DNA extracted using DNeasy Blood & Tissue Kit (Qiagen). Sequencing libraries were prepared by nested PCR method as described previously^63^ and were sequenced to a depth of 10^6^ reads with single-end 75 bp sequencing on a NextSeq (Illumina). Sequencing reads were demultiplexed using bcl2fastq (v2.17.1.14) and low-quality reads Q < 30 were removed. The reads were trimmed using cutadapt (v1.14)^64^ to extract the 20 bp targeting sequence and sgRNAs that were depleted relative to T0 determined using MAGeCK (v0.5.7)^65^. ggplot2 (v2.2.1) was used for figure generation.

### Quantitative RT-PCR

RNA was extracted from whole bone marrow using Trizol reagent and Directzol RNA miniprep kit (Zymo research) according to manufacturer’s instructions. cDNA was synthesised using High-Capacity cDNA Reverse Transcription Kit (Thermo Fisher) as per manufacturer’s instructions. qPCR was performed with SYBRgreen High-ROX mix (Agilent) on a LightCycler LC480 (Roche). Actin was used as a housekeeping control gene. Primer sequences are listed in Supplementary Table 3.

### Quant-seq

1–5 × 10^5^ sorted KL cells were lysed in Trizol reagent and RNA was extracted using Directzol RNA miniprep kit (Zymo research) according to manufacturer’s instructions. The Quant-seq 3′ mRNA-seq Library Prep Kit for Illumina (Lexogen) was used to generate libraries as per the manufacturer’s instructions. Libraries were pooled and sequenced with 75 bp single-end sequencing to a depth of 6–10 × 10^6^ reads on a NextSeq (Illumina). Sequencing reads were demultiplexed using bcl2fastq (v2.17.1.14) and low-quality reads Q < 30 were removed. The RNA sequencing reads were trimmed at the 5′ end using cutadapt (v1.14)^66^ to remove bias introduced by random primers used in the library preparation and 3′ end trimming was performed to eliminate poly-A-tail derived reads. Reads were mapped to the reference genome (mm10) using HISAT2. Reads were counted using subread software package (v1.5.0-p3)^63^. Differential gene expression analysis was performed using R package LIMMA (v3.32.4). R packages pheatmap (v1.0.8) and ggplot2 (v2.2.1) were used for figure generation. GSEA (v3.0) was used for analysing enrichment of gene sets and GO analysis was performed using metascape [http://metascape.org].

### ChIP-seq

KL and KSL cells were sorted from *Bcor*^*ΔE9-10*^ (*n* = 3), *Bcor*^*WT*^ (*n* = 2), *Bcor*^*ΔE9-10*^*Kras*^*G12D*^ (*n* = 3) and *Kras*^*G12D*^ (*n* = 3) mice. 1–5 × 10^5^ cells were crosslinked with 1% formaldehyde and 5% FCS in PBS at room temperature for 10 min, then the reactions were quenched with 1.25 M glycine for 5 min. Crosslinked cells were washed with ice cold dilution buffer (20 mM Tris pH 8, 150 mM NaCl,  2mM EDTA, 1%Triton-X) then resuspended in nuclear extraction buffer (50 mM TRIS pH7.5, 150 mM NaCl, 5 mM EDTA, 0.5%NP-40, 1% TritonX-100). Cells were washed in dilution buffer again before being lysed in ChIP lysis buffer (20 mM TRIS pH7.5, 150 mM NaCl, 0.5M EDTA, 1%NP-40, 0.3% SDS). Chromatin was fragmented to ~150–300 bp by sonicating lysates in a Covaris Ultrasonicator using 100 μl microtubules (Covaris). Immunoprecipitation was performed overnight at 4 °C in 50% dilution and 50% lysis buffer with magnetic protein A and G Dynabeads (Life technologies) pre-coupled to 1μg antibody per sample (antibodies listed in Supplementary Table 2). Beads were then washed with wash buffer 1 (20 mM TRIS pH 8, 500 mM NaCl, 2 mM EDTA, 1% Triton-X, 0.1% SDS) followed by wash buffer 2 (220 mM TRIS pH 8, 250 mM LiCl, 2 mM EDTA, 0.5% NP-40, 0.5% deoxycholate) and then TE buffer (10 mM TRIS pH7.5, 1 mM EDTA) on ice. Beads were then resuspended in reverse crosslinking buffer (1% SDS, 100 mM NaHCO3, 200 mM NaCL, 300 μg/mL proteinase K) and incubated at 55 °C for 30 min followed by 65 °C for 30 min. DNA was then purified using Zymo ChIP clean and concentrate kit according to manufacturer’s instructions (Zymo Research) and quantified using Qubit dsDNA HS Assay Kit (Thermo Fisher Scientific). Indexed libraries were prepared using KAPA Hyper Prep Kit for Illumina platforms (Kapa Biosystems) and the SeqCap Adapter Kit (Roche) following vendor’s instructions. Library QC and quantification was performed using D1000 high-sensitivity screen tape with 4200 TapeStation Instrument (Agilent Technologies) and size selected for between 200 bp and 500 bp using a Pippin Prep system (Sage Science). Libraries were pooled and sequenced with 75 bp single-end sequencing to a depth of 15–20 × 10^6^ reads per sample on a NextSeq (Illumina). bcl2fastq (v2.17.1.14) was used for de-multiplexing. The Fastq files generated by sequencing were aligned to the mouse reference genome (GRCm38/mm10) using bowtie2 (v2.3.3). Samtools (v1.4.1) was used for manipulation of SAM and BAM files. MACS (v2.1.1) was used for traditional peak-calling. R package Csaw (v1.10.0) was used to quantify regions enriched for histone marks, which were then associated with the closest TSS using Bedtools (v2.26). Superenhnacer analysis was performed using Homer (v4.8). Browser viewable TDF files were generated using IGVTools (v2.3.95) and ChIP-Seq tracks were visualized using IGV (v2.3.55). Graphics were generated using deeptools (v2.5.3) and R packages pheatmap (v1.0.8), plotly (v4.7.1) and ggplot2 (v2.2.1).

### ATAC-seq

ATAC-seq was performed using a previously published protocol^67^. In brief, 5 × 10^4^ sorted KL cells were lysed and nuclei washed, followed by chromatin tagmentation using TD1 enzyme and TDE buffer supplied in Illumina Nextera DNA prep kit (cat#FC-121–1030). Chromatin was tagmented at 37 °C for 30 min, then purified using the Minielute kit (Qiagen). A DNA library was prepared for sequencing by ligating adapters and amplifying using KAPA hot start polymerase (KAPA biosystems) according to manufacturer’s instructions then purifying using Minielute kit (Qiagen). QC was performed using D1000 high-sensitivity screen tape with 4200 TapeStation Instrument (Agilent Technologies) before size selection for fragments between 150 bp and 700 bp using a Pippin Prep System (Sage Science). Libraries were pooled and sequenced with 75bp single-end sequencing to a depth of 15–20 × 10^6^ reads per sample on a NextSeq (Illumina). bcl2fastq (v2.17.1.14) was used for de-multiplexing. The Fastq files generated by sequencing were aligned to the mouse reference genome (GRCm38/mm10) using bowtie2 (v2.3.3). Samtools (v1.4.1) was used for manipulation of SAM and BAM files. Data were visualised with deeptools (v2.5.3) alongside ChIP-seq data.

### Analysis of publically available data sets

H3K27ac and RNAseq BigWig files (From GSE60103^25^) were downloaded using SRA toolkit (v2.5.1), visualised in IgV (v2.4.5) and analysed alongside our ChIP-seq and Quant-seq data as above. GpG island data were downloaded from UCSC table browser. Data were converted using bedtools (v2.26) and ucsc-utils (v20161019) then analysed and visualised alongside our data with deeptools (v2.5.3) and IgV (v2.4.5).

For patient dataset #1 the series matrix file from Verhaak et al. (2009; GEO accession GSE6891^41^) was downloaded using the GEOquery R package (v2.42.0) and probe expression values were collapsed to median values per gene. Samples that had not been exome-sequenced were then removed from the analysis. For patient dataset #2 raw RNA counts and series matrix files were downloaded for the Leucegene project^39^ (GEO accessions GSE49642, GSE52656, GSE62190, GSE66917 and GSE67039). Counts were normalized with R using limma-voom (v3.32.10) and batch effects were removed using ComBat with the sva R package (v3.24.4). Differential expression analysis was performed using limma and results visualised using ggplot2 (v2.2.1). GSEA(v3.0) was used to compare to our RNA sequencing data above.

## Supplementary information

Supplementary Information

Description of Additional Supplementary Files

Supplementary Data 1

Supplementary Data 2

Supplementary Data 3

Supplementary Data 4

Supplementary Data 5

Supplementary Data 6

## Data Availability

The Quant-seq, ChIP-seq and ATAC-seq data generated in this study have been deposited into the Gene Expression Omnibus hosted at the National Center for Biotechnology under the accession number GSE126264. The authors declare that all data supporting the findings of this study are available within the article and its supplementary information or from the corresponding author upon reasonable request.

## References

[1] Ng D (2004). Oculofaciocardiodental and Lenz microphthalmia syndromes result from distinct classes of mutations in BCOR. Nat. Genet..

[2] Papaemmanuil E (2016). Genomic classification and prognosis in acute myeloid leukemia. N. Engl. J. Med..

[3] The Cancer Genome Atlas Research Network.. (2013). Genomic and epigenomic landscapes of adult de novo acute myeloid leukemia. N. Engl. J. Med..

[4] Grossmann V (2011). Whole-exome sequencing identifies somatic mutations of BCOR in acute myeloid leukemia with normal karyotype. Blood.

[5] Damm F (2013). BCOR and BCORL1 mutations in myelodysplastic syndromes and related disorders. Blood.

[6] Yoshizato T (2015). Somatic mutations and clonal hematopoiesis in aplastic anemia. N. Engl. J. Med..

[7] Béguelin W (2016). EZH2 and BCL6 cooperate to assemble CBX8-BCOR complex to repress bivalent promoters, mediate germinal center formation and lymphomagenesis. Cancer Cell..

[8] Cao Q (2016). BCOR regulates myeloid cell proliferation and differentiation. Leukemia.

[9] Lefebure M (2017). Genomic characterisation of Eμ-Myc mouse lymphomas identifies Bcor as a Myc co-operative tumour-suppressor gene. Nat. Commun..

[10] van den Boom V (2016). Non-canonical PRC1.1 targets active genes independent of H3K27me3 and is essential for leukemogenesis. Cell Rep..

[11] Tiberi L (2014). A BCL6/BCOR/SIRT1 complex triggers neurogenesis and suppresses medulloblastoma by repressing sonic hedgehog signaling. Cancer Cell..

[12] Koppens M, Van Lohuizen M (2016). Context-dependent actions of Polycomb repressors in cancer. Oncogene.

[13] Vidal M, Starowicz K (2017). Polycomb complexes PRC1 and their function in hematopoiesis. Exp. Hematol..

[14] Huynh KD, Fischle W, Verdin E, Bardwell VJ (2000). BCoR, a novel corepressor involved in BCL-6 repression. Genes Dev..

[15] Gearhart MD, Corcoran CM, Wamstad JA, Bardwell VJ (2006). Polycomb group and SCF ubiquitin ligases are found in a novel BCOR complex that is recruited to BCL6 targets. Mol. Cell. Biol..

[16] Gao Z (2012). PCGF homologs, CBX proteins, and RYBP define functionally distinct PRC1 family complexes. Mol. Cell.

[17] Wang Z (2018). A Non-canonical BCOR-PRC1. 1 complex represses differentiation programs in human ESCs. Cell. Stem. Cell..

[18] Oliviero G (2015). The variant polycomb repressor complex 1 component PCGF1 interacts with a pluripotency sub-network that includes DPPA4, a regulator of embryogenesis. Sci. Rep..

[19] Junco SE (2013). Structure of the polycomb group protein PCGF1 in complex with BCOR reveals basis for binding selectivity of PCGF homologs. Structure.

[20] Srinivasan RS, Erkenez de AC, Hemenway CS (2003). The mixed lineage leukemia fusion partner AF9 binds specific isoforms of the BCL-6 corepressor. Oncogene.

[21] Göthert JR (2005). In vivo fate-tracing studies using the Scl stem cell enhancer: embryonic hematopoietic stem cells significantly contribute to adult hematopoiesis. Blood.

[22] Shih AH (2015). Mutational cooperativity linked to combinatorial epigenetic gain of function in acute myeloid leukemia. Cancer Cell..

[23] Hnisz D (2013). Transcriptional super-enhancers connected to cell identity and disease. Cell.

[24] Whyte WA (2013). Master transcription factors and mediator establish super-enhancers at key cell identity genes. Cell.

[25] Lara-Astiaso D (2014). Chromatin state dynamics during blood formation. Science.

[26] Lun AT, Smyth GK (2015). csaw: a Bioconductor package for differential binding analysis of ChIP-seq data using sliding windows. Nucleic Acids Res..

[27] Wu X, Johansen JV, Helin K (2013). Fbxl10/Kdm2b recruits polycomb repressive complex 1 to CpG islands and regulates H2A ubiquitylation. Mol. Cell.

[28] Thorsteinsdottir U (2002). Overexpression of the myeloid leukemia-associated Hoxa9 gene in bone marrow cells induces stem cell expansion. Blood.

[29] Afonja O (2000). MEIS1 and HOXA7 genes in human acute myeloid leukemia. Leuk. Res..

[30] Dillon LM (2015). P-REX1 creates a positive feedback loop to activate growth factor receptor, PI3K/AKT and MEK/ERK signaling in breast cancer. Oncogene.

[31] Shukla A (2014). CLIC4 regulates TGF-beta-dependent myofibroblast differentiation to produce a cancer stroma. Oncogene.

[32] Vignudelli T (2010). ZFP36L1 negatively regulates erythroid differentiation of CD34+ hematopoietic stem cells by interfering with the Stat5b pathway. Mol. Biol. Cell..

[33] Rust R (2005). High expression of calcium-binding proteins, S100A10, S100A11 and CALM2 in anaplastic large cell lymphoma. Br. J. Haematol..

[34] Blackledge NP (2014). Variant PRC1 complex-dependent H2A ubiquitylation drives PRC2 recruitment and polycomb domain formation. Cell.

[35] Lu R (2016). Epigenetic perturbations by Arg882-mutated DNMT3A potentiate aberrant stem cell gene-expression program and acute leukemia development. Cancer Cell..

[36] Metzeler KH (2016). Spectrum and prognostic relevance of driver gene mutations in acute myeloid leukemia. Blood.

[37] Haferlach T (2014). Landscape of genetic lesions in 944 patients with myelodysplastic syndromes. Leukemia.

[38] Johnson L (2001). Somatic activation of the K-ras oncogene causes early onset lung cancer in mice. Nature.

[39] Lavallée VP (2015). The transcriptomic landscape and directed chemical interrogation of MLL-rearranged acute myeloid leukemias. Nat. Genet..

[40] Glass J (2017). Epigenetic identity in AML depends on disruption of non-promoter regulatory elements and is affected by antagonistic effects of mutations in epigenetic modifiers. Cancer Discov..

[41] Verhaak RGW (2009). Prediction of molecular subtypes in acute myeloid leukemia based on gene expression profiling. Haematologica.

[42] Brunetti L (2018). Mutant NPM1 maintains the leukemic state through HOX expression. Cancer Cell..

[43] Rice KL, Licht JD (2007). HOX deregulation in acute myeloid leukemia. J. Clin. Invest..

[44] Challen GA (2011). Dnmt3a is essential for hematopoietic stem cell differentiation. Nat. Genet..

[45] Faber J (2009). HOXA9 is required for survival in human MLL-rearranged acute leukemias. Blood.

[46] McCormack MP (2010). The Lmo2 oncogene initiates leukemia in mice by inducing thymocyte self-renewal. Science.

[47] Kesarwani M (2017). Targeting c-FOS and DUSP1 abrogates intrinsic resistance to tyrosine-kinase inhibitor therapy in BCR-ABL-induced leukemia. Nat. Med..

[48] Lord KA, Abdollahi A, Hoffman-Liebermann B, Liebermann DA (1993). Proto-oncogenes of the fos/jun family of transcription factors are positive regulators of myeloid differentiation. Mol. Cell. Biol..

[49] Mikkola HKA (2003). Haematopoietic stem cells retain long-term repopulating activity and multipotency in the absence of stem-cell leukaemia SCL/tal-1 gene. Nature.

[50] Shivdasani RA, Mayer EL, Orkin SH (1995). Absence of blood formation in mice lacking the T-cell leukaemia oncoprotein tal-1/SCL. Nature.

[51] Condorelli GL (1996). T-cell-directed TAL-1 expression induces T-cell malignancies in transgenic mice. Cancer Res..

[52] Sánchez C (2007). Proteomics analysis of Ring1B/Rnf2 interactors identifies a novel complex with the Fbxl10/Jhdm1B histone demethylase and the Bcl6 interacting corepressor. Mol. Cell. Proteom..

[53] Rossi A (2016). Maintenance of leukemic cell identity by the activity of the Polycomb complex PRC1 in mice. Sci. Adv..

[54] Klauke K (2013). Polycomb Cbx family members mediate the balance between haematopoietic stem cell self-renewal and differentiation. Nat. Cell Biol..

[55] Zhang XW (2010). BMI1 and Mel-18 oppositely regulate carcinogenesis and progression of gastric cancer. Mol. Cancer.

[56] Guo WJ (2007). Mel-18 acts as a tumor suppressor by repressing Bmi-1 expression and down-regulating Akt activity in breast cancer cells. Cancer Res..

[57] Jacobs JJ (1999). Bmi-1 collaborates with c-Myc in tumorigenesis by inhibiting c-Myc-induced apoptosis via INK4a/ARF. Genes Dev..

[58] Bernard D (2005). CBX7 controls the growth of normal and tumor-derived prostate cells by repressing the Ink4a/Arf locus. Oncogene.

[59] Kroon E (1998). Hoxa9 transforms primary bone marrow cells through specific collaboration with Meis1a but not Pbx1b. EMBO J..

[60] Zekavati A (2014). Post-transcriptional regulation of BCL2 mRNA by the RNA-binding protein ZFP36L1 in malignant B cells. PLoS ONE.

[61] Tanaka T (2017). Internal deletion of BCOR reveals a tumor suppressor function for BCOR in T lymphocyte malignancies. J. Exp. Med..

[62] Doench JG (2016). Optimized sgRNA design to maximize activity and minimize off-target effects of CRISPR-Cas9. Nat. Biotechnol..

[63] Joung J (2017). Genome-scale CRISPR-Cas9 knockout and transcriptional activation screening. Nat. Protoc..

[64] Martin, M. Cutadapt removes adapter sequences from high-throughput sequencing reads. *EMBnet. J* **17**, 10–12 (2011).

[65] Li W (2014). MAGeCK enables robust identification of essential genes from genome-scale CRISPR/Cas9 knockout screens. Genome Biol..

[66] Liao Y, Smyth GK, Shi W (2013). The Subread aligner: fast, accurate and scalable read mapping by seed-and-vote. Nucleic Acids Res..

[67] Buenrostro JD, Giresi PG, Zaba LC, Chang HY, Greenleaf WJ (2013). Transposition of native chromatin for fast and sensitive epigenomic profiling of open chromatin, DNA-binding proteins and nucleosome position. Nat. Methods.

[68] Kanamori M (2004). A genome-wide and nonredundant mouse transcription factor database. Biochem. Biophys. Res. Commun..

[69] Waardenberg AJ, Bassett SD, Bouveret R, Harvey RP (2015). CompGO: an R package for comparing and visualizing Gene Ontology enrichment differences between DNA binding experiments. BMC Bioinforma..

