# Supplementary material for: Bcor loss perturbs myeloid differentiation and promotes leukaemogenesis
Source: Nat Commun. 2019 Mar 22;10:1347. doi: 10.1038/s41467-019-09250-6 (PMC6430802; doi:10.1038/s41467-019-09250-6)
Supplement: Supplementary file 1 — Supplementary Information [file 41467_2019_9250_MOESM1_ESM.pdf]

Bcor loss perturbs myeloid differentiation and promotes leukaemogenesis

Supplementary Information

Kelly et al.

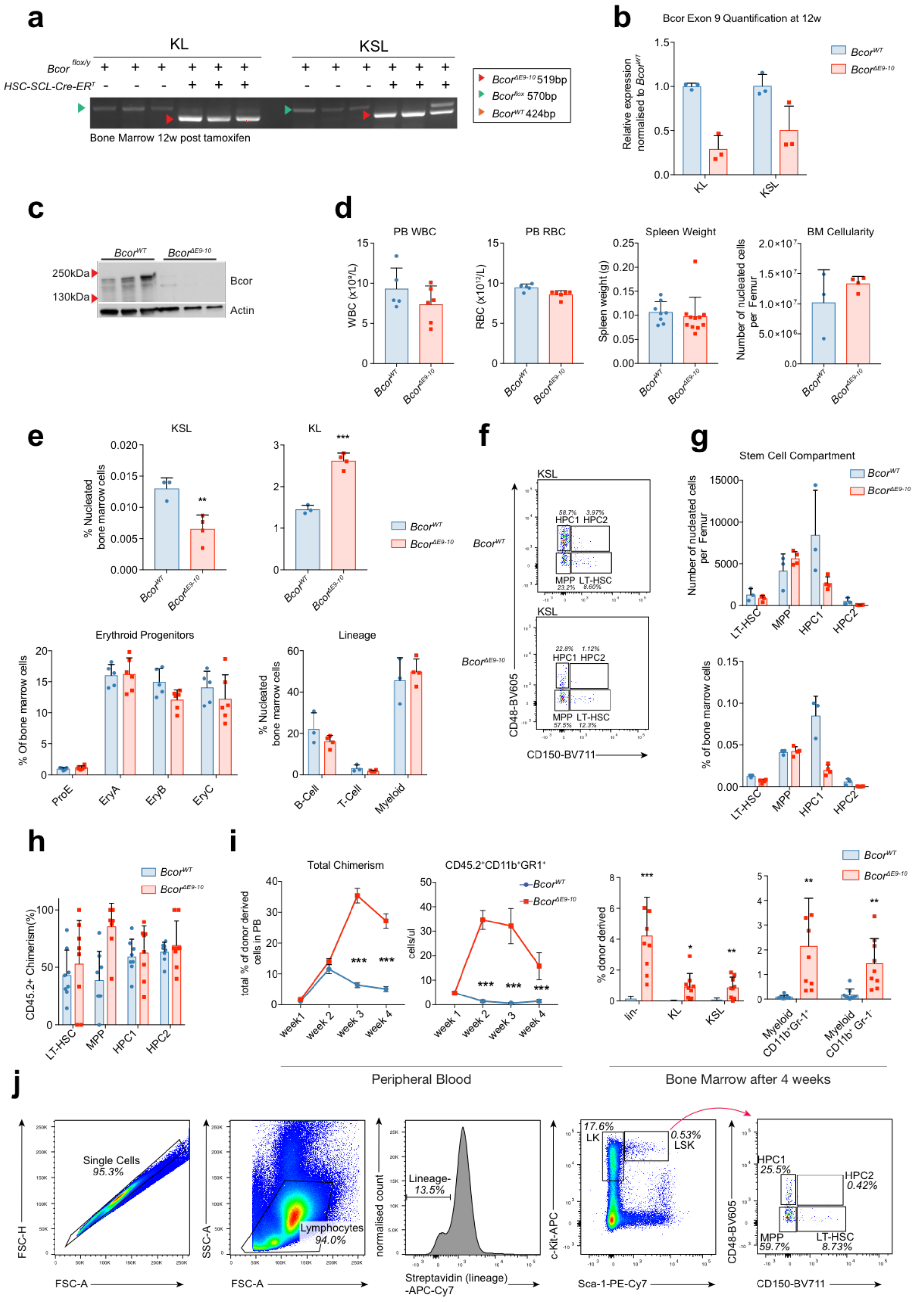
a
KL
KSL
Bcor flox/y
HSC-SCL-Cre-ERT
Bone Marrow 12w post tamoxifen
BcorΔE9-10 519bp
Bcorflox 570bp
BcorWT 424bp
b
Bcor Exon 9 Quantification at 12w
Relative expression normalised to BcorWT
BcorWT
BcorΔE9-10
c
250kDa
130kDa
Bcor
Actin
d
PB WBC
WBC (x10⁹/L)
PB RBC
RBC (x10¹²/L)
Spleen Weight
Spleen weight (g)
BM Cellularity
Number of nucleated cells per Femur
e
KSL
KL
% Nucleated bone marrow cells
Erythroid Progenitors
% Of bone marrow cells
ProE
EryA
EryB
EryC
Lineage
B-Cell
T-Cell
Myeloid
f
CD48-BV605
CD150-BV711
HPC1
HPC2
MPP
LT-HSC
g
Stem Cell Compartment
Number of nucleated cells per Femur
% of bone marrow cells
h
CD45.2+ Chimerism(%)
i
Total Chimerism
total % of donor derived cells in PB
CD45.2+CD11b+GR1+
cells/ul
week 1
week 2
week 3
week 4
% donor derived
lin-
Myeloid CD11b+Gr-1+
Myeloid CD11b+ Gr-1-
Peripheral Blood
Bone Marrow after 4 weeks
j
Single Cells
95.3%
FSC-H
FSC-A
Lymphocytes
94.0%
SSC-A
Lineage-
13.5%
normalised count
Streptavidin (lineage) -APC-Cy7
17.6%
LK
0.53%
LSK
c-Kit-APC
Sca-1-PE-Cy7
HPC1
25.5%
HPC2
0.42%
MPP
59.7%
LT-HSC
8.73%

Supplementary Figures

Supplementary figure 1. ***Bcor* regulates myeloid differentiation *in vivo*, related to figure 1.** (a) Deletion of *Bcor* exons 9 and 10 was confirmed in sorted KL and KSL populations from $Bcor^{flox}$;$HSC\text{-}SCL\text{-}Cre\text{-}ER^{T+}$ and $Bcor^{flox}$;$HSC\text{-}SCL\text{-}Cre\text{-}ER^{T-}$ mice 12 weeks post tamoxifen administration by PCR genotyping. (b) Recombination at exon 9 of the *Bcor* gene was quantified by qPCR using primers internal to the floxed region (see Supplementary Table 1) and normalized to *Bcor* intron 6. (n=3, KL p=9.1x10^{-4}, KSL p=6.8 x10^{-3}). (c) Western blot for Bcor in *ex vivo* cultured KL cells from 3 independent $Bcor^{WT}$ and $Bcor^{\Delta E9\text{-}10}$ mice. (d) Average values for spleen weights (n=10), peripheral white blood cells and red blood cells (n=5), and total bone marrow cellularity (n=3) for $Bcor^{WT}$ (in blue) and $Bcor^{\Delta E9\text{-}10}$ (in red) mice at 20 weeks post tamoxifen administration. (e) Average percentage of cell populations within bone marrow mononuclear cells from one femur from $Bcor^{WT}$ and $Bcor^{\Delta E9\text{-}10}$ mice (n = 5, means were compared with unpaired t-test, KSL p = 4.8 x10^{-3}, KL P =1.91 x10^{-4}). (f) Representative flow cytometry plot of KSL sub-populations from the BM of $Bcor^{WT}$ and $Bcor^{\Delta E9\text{-}10}$ mice. (g) Average absolute numbers of stem cell populations quantified by flow cytometry. Each replicate represents the absolute number within the bone marrow of a single femur of a single mouse (n=5, means were compared with unpaired t-Test, LT-HSC p=9.3x10^{-3}, HPC1 p=2.7 x10^{-3}). (h) Average percentage of donor-derived cells ($CD45.2^{+}$) in the indicated BM populations in recipients transplanted with whole bone marrow mononuclear cells (n=8, means were compared with unpaired t-test, N.S.). (i) Chimerism and absolute numbers of donor-derived cells in the peripheral blood or bone marrow of recipient mice transplanted with sorted KL cells (n=8; means compared with unpaired t-test: PB week3, p=2.70 x10^{-9}, week 4, p=1.47 x10^{-7}, BM chimerism lin^{-} p=1.5x10^{-4}, KL p=5.0x10^{-3}, KSL p=2.4 x10^{-3}, Myeloid $CD11b^{+}$only p=2.2 x10^{-3}, myeloid $CD11b^{+}Gr1^{+}$ p =5.4 x10^{-3}). (j) Example flow cytometry gating strategy used for immunophenotyping analysis and FACS isolation of KL cells. For all bar graphs values from individual animals are shown, bars indicate mean ± standard deviation.

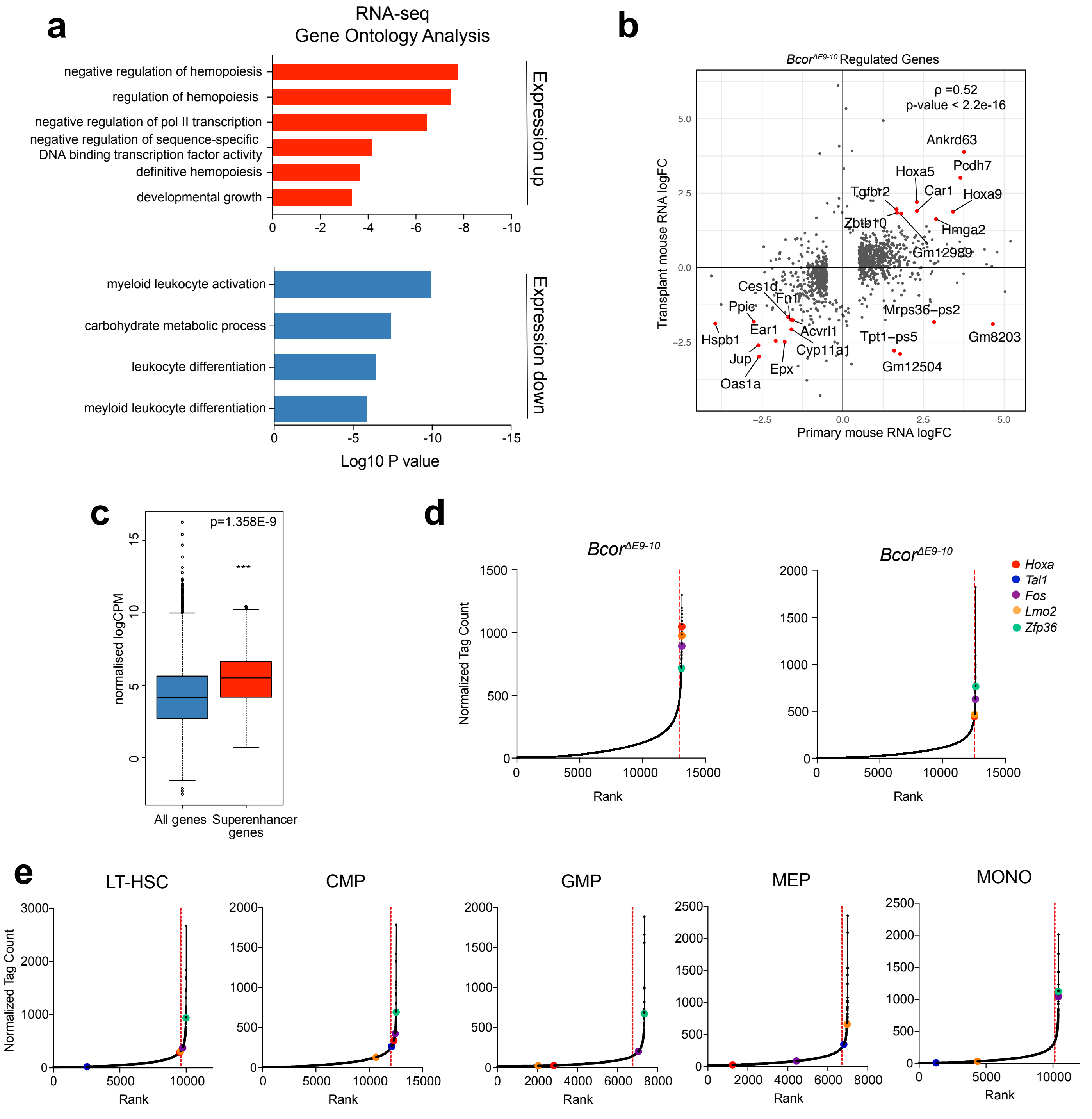

Supplementary figure 2. **Bcor regulates HSC associated master transcription factor genes during myeloid differentiation, related to figure 2.** (a) GO terms enrichment analysis on genes that are significantly differentially expressed (p<0.05) between *Bcor*$^{\Delta E9-10}$ and *Bcor*$^{\Delta E9-10}$ KL cells. (b) Correlation between gene expression changes in KL cells sorted from primary transgenic mice (horizontal) or transplanted recipients (vertical). Plotted are genes for which log_2FC is >0.5 or <-0.5 in either experiment and highlighted are genes for which log_2FC is >1 or <-1 and p<0.01 in either condition. Correlation analysis performed with Spearmans

rank correlation (ρ =0.52, p-value < 2.2 x10^{-16}). (c) Tukey boxplot of RNA expression for genes associated with super-enhancers compared with all expressed genes in *Bcor*$^{\Delta E9-1}$ KL cells. Centre line denotes median, whiskers extend to 1.5xIQR. Comparison of means was performed with Welch two sample t-test (t=6.5126, df = 134.37, p = 1.385 x10^{-9}). (d) All enhancers identified in *Bcor*$^{\Delta E9-10}$ samples (total n=3) and (e) hematopoietic cell populations, ranked by tag count. Red dashed line indicates a super-enhancer slope of 1. Highlighted are genes identified in Fig. 2D.

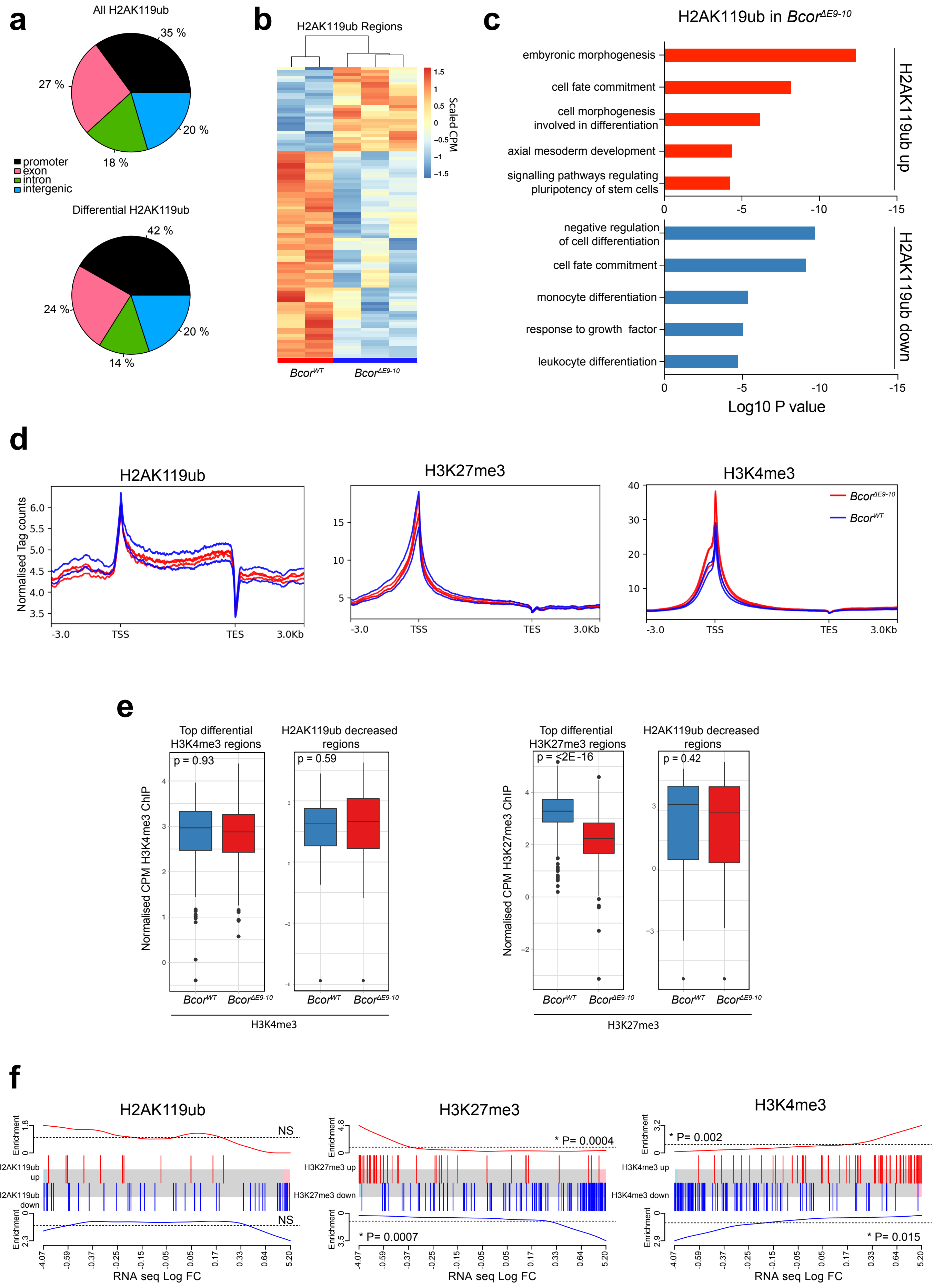
a
All H2AK119ub
35 %
27 %
20 %
18 %
promoter
exon
intron
intergenic
Differential H2AK119ub
42 %
24 %
20 %
14 %
b
H2AK119ub Regions
Scaled CPM
1.5
1
0.5
0
−0.5
−1
−1.5
Bcor^WT
Bcor^ΔE9-10
c
H2AK119ub in Bcor^ΔE9-10
embryonic morphogenesis
cell fate commitment
cell morphogenesis involved in differentiation
axial mesoderm development
signalling pathways regulating pluripotency of stem cells
H2AK119ub up
negative regulation of cell differentiation
cell fate commitment
monocyte differentiation
response to growth factor
leukocyte differentiation
H2AK119ub down
0
-5
-10
-15
Log10 P value
d
H2AK119ub
H3K27me3
H3K4me3
Normalised Tag counts
-3.0
TSS
TES
3.0Kb
Bcor^ΔE9-10
Bcor^WT
e
Top differential H3K4me3 regions
p = 0.93
H2AK119ub decreased regions
p = 0.59
Normalised CPM H3K4me3 ChIP
Bcor^WT
Bcor^ΔE9-10
H3K4me3
Top differential H3K27me3 regions
p = <2E-16
H2AK119ub decreased regions
p = 0.42
Normalised CPM H3K27me3 ChIP
H3K27me3
f
H2AK119ub
NS
H2AK119ub up
H2AK119ub down
NS
Enrichment
H3K27me3
* P= 0.0004
H3K27me3 up
H3K27me3 down
* P= 0.0007
H3K4me3
* P= 0.002
H3K4me3 up
H3K4me3 down
* P= 0.015
-4.07
-0.59
-0.37
-0.25
-0.15
-0.05
0.05
0.17
0.33
0.64
5.20
RNA seq Log FC

Supplementary figure 3. **Bcor regulates H2AK119ub at a small subset of H2AK119ub-asscoiated loci, related to figure 3.** (a) Genomic location of all H2AK119ub-enriched regions in *Bcor*^{WT} KL cells and differentially ubiquitinated regions between *Bcor*^{WT} and *Bcor*$^{\Delta E9\text{-}10}$ KL cells. (b) Heatmap of H2AK119ub ChIP-seq signal from the top 500 differentially ubiquitinated regions between *Bcor*^{WT} and *Bcor*$^{\Delta E9\text{-}10}$ KL cells. (c) GO term enrichment analysis for genes associated with differentially ubiquitinated regions between *Bcor*^{WT} and *Bcor*$^{\Delta E9\text{-}10}$ KL cells (H2AK119ub up in *Bcor*$^{\Delta E9\text{-}10}$, $log_2FC>0.5$ and $p<0.05$; H2AK119ub down in *Bcor*$^{\Delta E9\text{-}10}$, $log_2FC<-0.5$ and $p<0.05$). (d) ChIP-seq metagene plots of H2AK119ub, H3K27me3 and H3K4me3 enrichment ± 3kb flanking scaled genes. Each line represents one ChIP-seq replicate. (e) Tukey boxplots normalized signal from ChIP–seq experiments. H3K27me3 and H3K4me3 signal was quantified in the top 100 regions significantly differentially enriched for each mark, as well as in regions with a significant loss of ubiquitin. Means compared with unpaired t-test. Centre line denotes median, whiskers extend to 1.5xIQR. (f) Barcode plots showing association between mRNA expression and changes in histone marks (red, histone mark significantly increased $log_2FC>0.5$ and $p<0.05$; blue, histone mark significantly decreased $log_2FC<-0.5$ and $p<0.05$). Significant enrichment for gene sets was tested with rotation gene set tests (ROAST)[1], p values indicated.

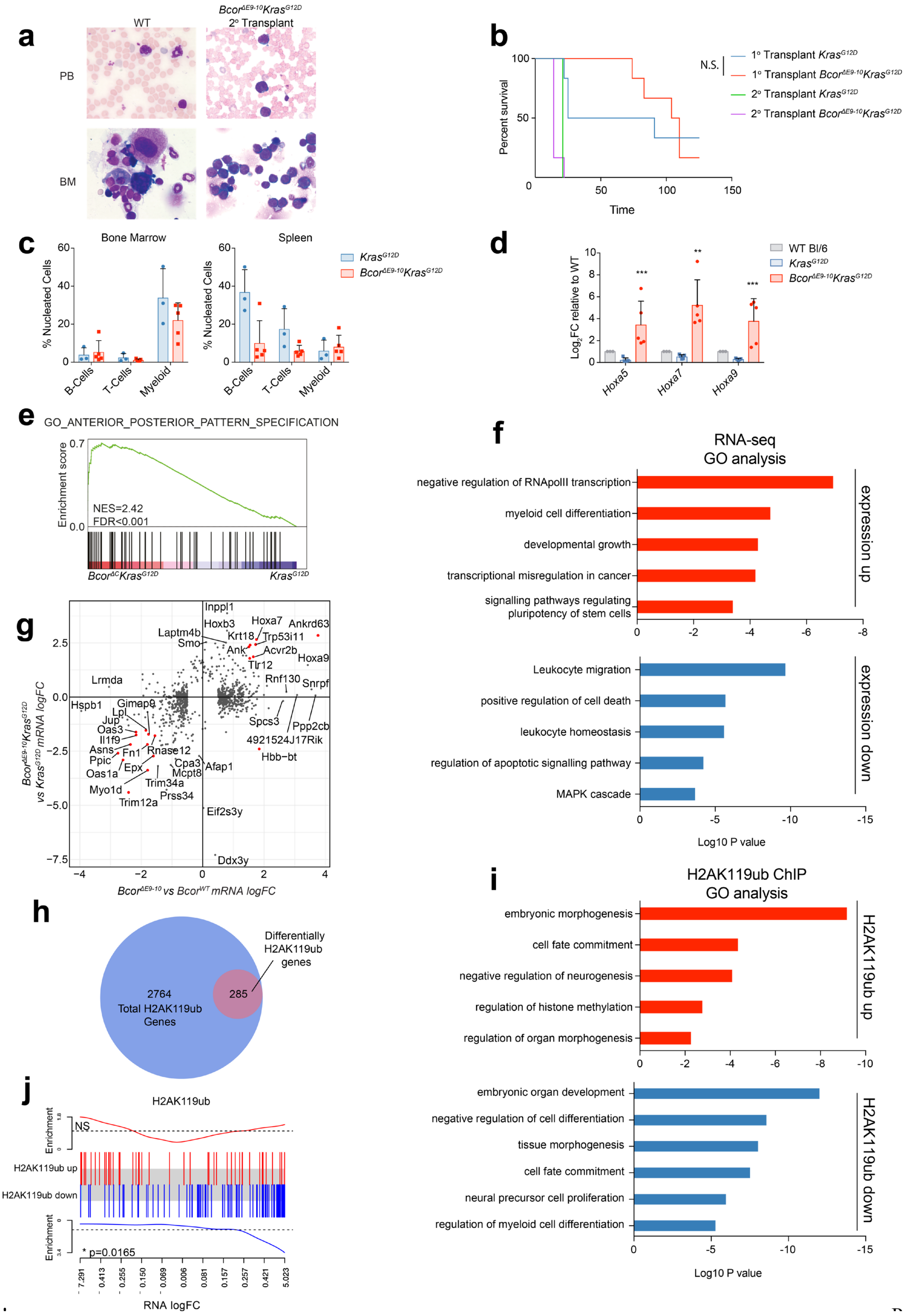
a
WT
BcorΔE9-10KrasG12D 2° Transplant
PB
BM
b
Percent survival
Time
N.S.
1° Transplant KrasG12D
1° Transplant BcorΔE9-10KrasG12D
2° Transplant KrasG12D
2° Transplant BcorΔE9-10KrasG12D
c
Bone Marrow
Spleen
% Nucleated Cells
B-Cells
T-Cells
Myeloid
KrasG12D
BcorΔE9-10KrasG12D
d
Log2FC relative to WT
WT Bl/6
KrasG12D
BcorΔE9-10KrasG12D
Hoxa5
Hoxa7
Hoxa9
e
GO_ANTERIOR_POSTERIOR_PATTERN_SPECIFICATION
Enrichment score
NES=2.42
FDR<0.001
BcorΔCKrasG12D
KrasG12D
f
RNA-seq GO analysis
negative regulation of RNApolII transcription
myeloid cell differentiation
developmental growth
transcriptional misregulation in cancer
signalling pathways regulating pluripotency of stem cells
expression up
Leukocyte migration
positive regulation of cell death
leukocyte homeostasis
regulation of apoptotic signalling pathway
MAPK cascade
expression down
Log10 P value
g
BcorΔE9-10KrasG12D vs KrasG12D mRNA logFC
BcorΔE9-10 vs BcorWT mRNA logFC
Inppl1
Hoxb3
Hoxa7
Ankrd63
Laptm4b
Smo
Krt18
Trp53i11
Ank
Acvr2b
Hoxa9
Tlr12
Lrmda
Rnf130
Snrpf
Hspb1
Gimap9
Lpl
Jup
Oas3
Il1f9
Asns
Fn1
Rnase12
Ppic
Epx
Cpa3
Oas1a
Mcpt8
Afap1
Trim34a
Myo1d
Prss34
Trim12a
Spcs3
Ppp2cb
4921524J17Rik
Hbb−bt
Eif2s3y
Ddx3y
h
Differentially H2AK119ub genes
2764 Total H2AK119ub Genes
285
i
H2AK119ub ChIP GO analysis
embryonic morphogenesis
cell fate commitment
negative regulation of neurogenesis
regulation of histone methylation
regulation of organ morphogenesis
H2AK119ub up
embryonic organ development
negative regulation of cell differentiation
tissue morphogenesis
cell fate commitment
neural precursor cell proliferation
regulation of myeloid cell differentiation
H2AK119ub down
Log10 P value
j
H2AK119ub
Enrichment
NS
H2AK119ub up
H2AK119ub down
* p=0.0165
RNA logFC

Supplementary figure 4. **$Bcor^{\Delta E9\text{-}10}$ cooperates with $Kras^{G12D}$ to initiate leukaemia and regulate transcription and H2AK119ub, related to figure 4.** (a) Representative images of Romanowsky staining peripheral blood smears (PB) and bone marrow cytospins (BM) from C57BL/6 (WT) mice or secondary recipients transplanted with $Bcor^{\Delta E9\text{-}10}Kras^{G12D}$ leukaemic cells (clone #72) illustrating lack of tri-lineage differentiation and accumulation of blasts in both PB and BM. (b) Kaplan-Meier survival curve illustrating survival of mice serially transplanted with $Bcor^{\Delta E9\text{-}10}Kras^{G12D}$ or $Kras^{G12D}$ tumours. 1st transplant n=6 mice pooled from 2 clones, 2nd transplant n=6 mice, one clone from each genotype. Statistical comparison of curves performed with Gehan-Breslow-Wilcoxin test. (c) Percentage of nucleated cells in either the bone marrow or spleen of moribund $Bcor^{\Delta E9\text{-}10}Kras^{G12D}$ or $Kras^{G12D}$ mice positive for lineage markers measured by flow cytometry. ($Bcor^{\Delta E9\text{-}10}Kras^{G12D}$ n = 5 or $Kras^{G12D}$ n = 3 mice). (d) *Hoxa5, 7* and *9* mRNA expression in whole bone marrow of moribund $Bcor^{\Delta E9\text{-}10}Kras^{G12D}$ and $Kras^{G12D}$ mice measured by qPCR, log fold change values relative to $Bcor^{WT}$ control, and normalized to *Actin*. 5 biological replicates for $Bcor^{\Delta E9\text{-}10}Kras^{G12D}$ and $Kras^{G12D}$ and 1 for $Bcor^{WT}$ are shown and 3 technical replicates were performed. (e) GSEA enrichment plots comparing Quant-seq from $Bcor^{\Delta E9\text{-}10}Kras^{G12D}$ and $Kras^{G12D}$ KL cells against a HOX-associated gene set. (f) GO term enrichment analysis for genes differentially expressed between $Bcor^{\Delta E9\text{-}10}Kras^{G12D}$ and $Kras^{G12D}$ KL cells log_2FC is >0.5 or <-0.5 and p<0.05) in $Bcor^{\Delta E9\text{-}10}Kras^{G12D}$ compared to $Kras^{G12D}$ only. (g) Correlation between gene expression changes in normal (comparing $Bcor^{\Delta E9\text{-}10}$ and $Bcor^{WT}$ KL cells, horizontal) and malignant (comparing $Bcor^{\Delta E9\text{-}10}Kras^{G12D}$ and $Kras^{G12D}$ KL cells, vertical) contexts. Plotted are genes for which log_2FC is >0.5 or <-0.5 and p<0.01 in either experiment, highlighted in red are genes for which log_2FC is >1.5 or <-1.5 and annotated are genes for which log_2FC is >2.5 or <-2.5 either condition. Correlation analysis performed with Pearson's product-moment correlation (ρ = 0.3589, p<2.2e-16). (h) Venn diagram overlaying all genes associated with H2AK119ub-enriched regions in KL cells and genes associated with differentially ubiquitinated regions in $Bcor^{\Delta E9\text{-}10}Kras^{G12D}$ KL cells compared with $Kras^{G12D}$ KL cells. (i) GO term enrichment analysis for genes that which are differentially ubiquitinated (log_2FC>0.3 or <-0.3) in $Bcor^{\Delta E9\text{-}10}Kras^{G12D}$ compared to $Kras^{G12D}$ KL cells. (j) Barcode plot demonstrating enrichment of differentially expressed genes within genes associated with either significant increase (red) or decrease (blue) in H2AK119ub. Enrichment for gene sets was tested with ROAST[1]. Genes associated with an increase in

H2AK119ub signal (logFC>0.3, p<0.05) were not enriched in gene expression (NS), whilst genes with decreased H2AK119ub (logFC<-0.3, p<0.05) were significantly enriched in differentially expressed genes in $Bcor^{\Delta E9\text{-}10}Kras^{G12D}$ (p=0.0165). Mean values are shown in all figure bargraphs and error bars represent standard deviation.

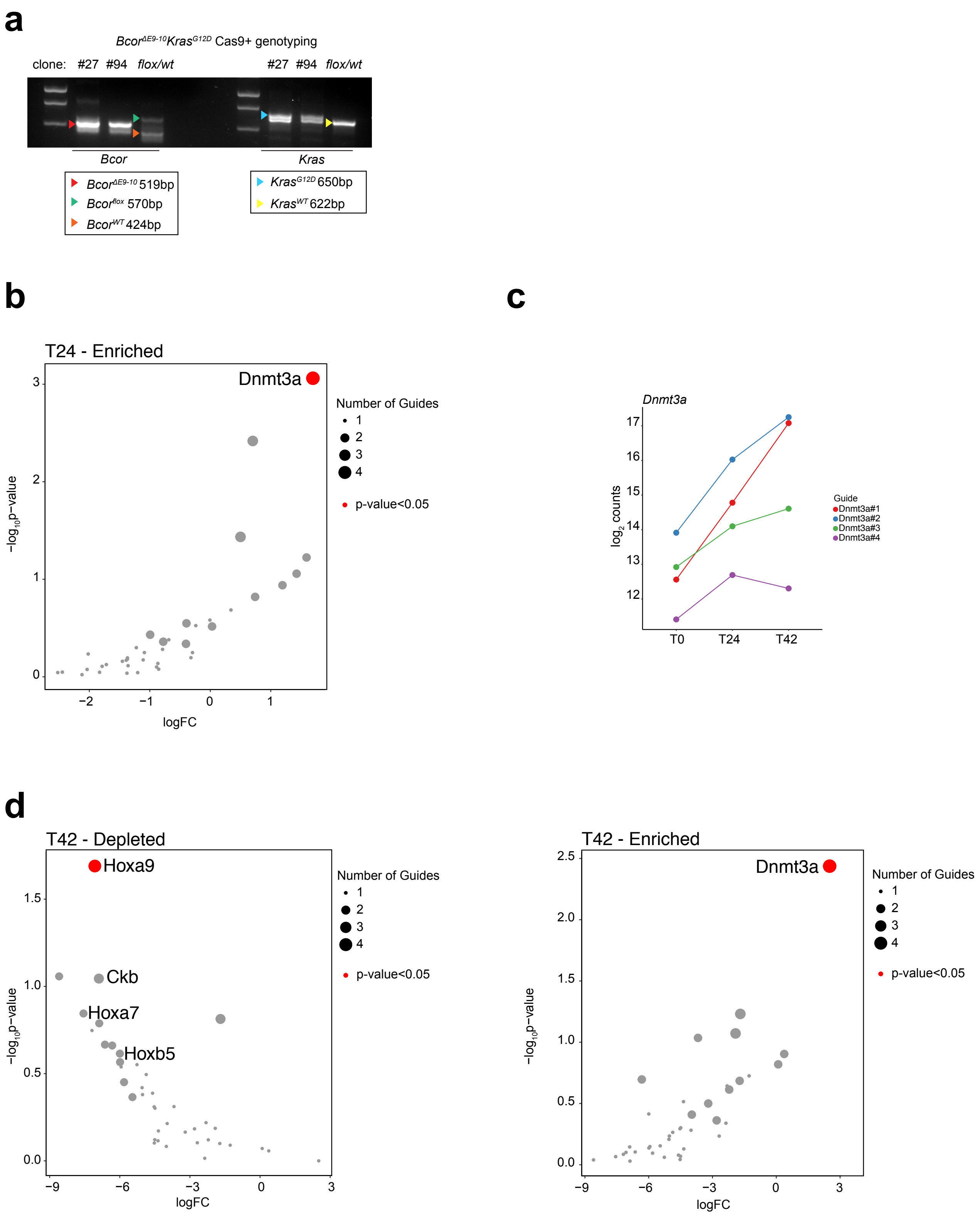

Supplementary figure 5. **CRISPR screen in *Bcor*$^{\Delta E9\text{-}10}$*Kras*^{G12D} leuakemic cells.** (a) Genotyping results of *Bcor*$^{\Delta E9\text{-}10}$*Kras*^{G12D} tumour cells utilized for the screen cultured *ex vivo* and *Bcor*$^{flox/wt}$ control DNA. (b) Positive enrichment of all genes in the CRISPR screen at T24 calculated by MAGeCK. (c) Dnmt3a-targeting sgRNA counts at T0, T24 and T42. (d) Negative and positive enrichment of all genes in the CRISPR screen at T42 calculated by MAGeCK.

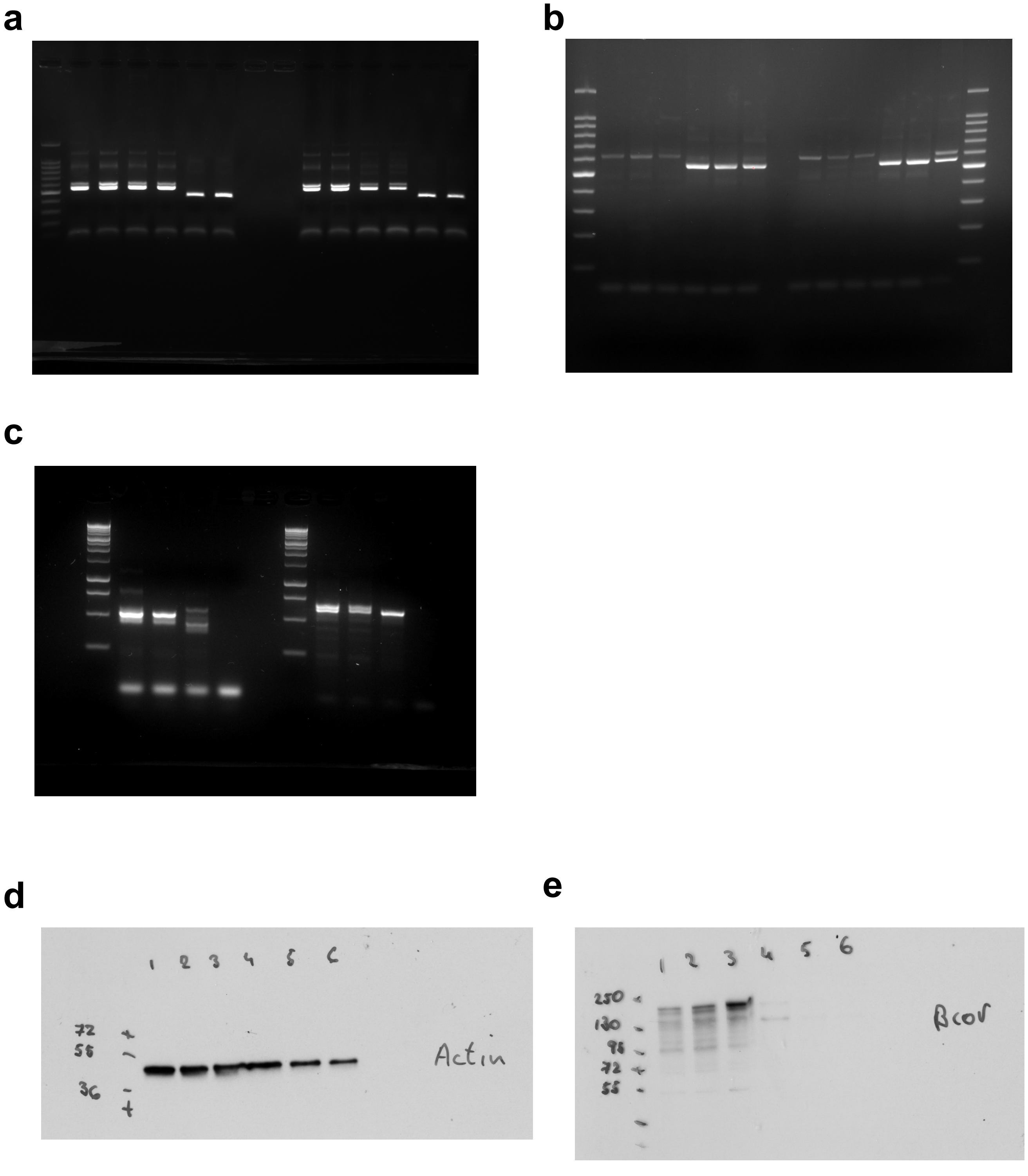

Supplementary figure 6. **Full, uncropped images of gels and blots.** (a) Genotyping DNA agarose gel, as related to Fig. 1B. lane 1: 100bp DNA Ladder, lane 2-5: 20w $Bcor^{\Delta E9\text{-}10}$ KL cells, lane 6-7: 20w $Bcor^{WT}$ KL cells, lane 10-13: 20w $Bcor^{\Delta E9\text{-}10}$ Lin+ cells, lane 14-15: 20w $Bcor^{WT}$ Lin+ cells. (b) Genotyping DNA agarose gel – as related to Supplementary Fig. 1A. lane 1: 100bp DANA Ladder, lane 2-4: $Bcor^{\Delta E9\text{-}10}$ KL cells, lane 5-7: $Bcor^{WT}$ KL cells, lane 9-11: $Bcor^{\Delta E9\text{-}10}$ KSL cells, lane 12-14: $Bcor^{WT}$ KSL cells, lane 15: 100bp DNA ladder. (c) Genotyping DNA agarose gel, as related to Fig. 4A. lane 1: 100bp DNA ladder, lane 2: BK clone#27 Bcor

genotyping, lane 3 BK clone 94 Bcor genotyping, $Bcor^{flox/WT}$ control DNA with Bcor genotyping, lane 5; 100bp DNA ladder, lane 6: BK clone#27 Kras genotyping, lane 3: BK clone 94 Kras genotyping, $Bcor^{flox/WT}$ control DNA with Kras genotyping. (d) Western Blot – as related to Supplementary figure 1C. Bcor western blot performed as described in methods with antibodies described in Supplementary Table 2. Lane 1-3: $Bcor^{WT}$ KL, lane 4-6: $Bcor^{\Delta E9\text{-}10}$ KL. Molecular weight labelled. (e) Western Blot, as related to Supplementary Fig. 1C. Actin western blot performed with Ab in Supplementary Table 2. Lane 1-3: $Bcor^{WT}$ KL, lane 4-6: $Bcor^{\Delta E9\text{-}10}$ KL.

Supplementary Tables

Supplementary Table 1: Flow cytometry antibodies

Antibody	Species	Fluorophore	Source	Cat. No.
cKIT/CD117	Mouse	APC	BD Pharmingen	553356
Sca-1/ ly6a/e	Mouse	PE-Cy7	BD Pharmingen	558162
Fcy II/III	Mouse	PE	BD Pharmingen	553145
CD34	Mouse	BV 421	BD Pharmingen	562608
CD150	Mouse	BV711	Biolegend	115941
CD48	Mouse	BV605	Biolegend	103441
CD11b/Mac-1	Mouse	V450	BD Biosciences	560455
B220	Mouse	APC	eBioscience	17-0452-83
CD3	Mouse	PE	BD Pharmingen	555275
Gr-1/ly6C/G	Mouse	alexaFluor700	BioLegend	108422
CD45.2	Mouse	FitC	BD Biosciences	553772
lineage	Mouse	Biotin	Miltenyi Biotech	130-090-858
Anti-biotin	Mouse	APC-Cy7	Biolegend	405208

Supplementary Table 2: ChIP and western blot antibodies

Antibody	Species	Source	Cat. No.	Clone/lot no.
H2AK119ub	Mouse/human	Cell signalling technology	8240S	2
H3K27me3	Mouse/human	Millipore	07449	2736613
H3K27ac	Mouse/human	Abcam	AB4729	323154-1
H3K4Me3	Mouse/human	Abcam	AB8580	GR273043
Bcor	Mouse/human	Vivian Barwell[2,3]		
β-actin	Mouse/human	Sigma-Aldrich	A2228	
2º - anti Rabbit IGG/HRP	rabbit	Dako	P0217	20047666
2º - anti mouse IgG/HRP	mouse	Dako	P0260	20030273

Supplementary Table 3: Primers

**Name**	**Use**	**Sequence**	
C2	Genotyping	GTATGCAGAGACCACCTCTTGGC	
G	Genotyping	ACGGTACCGTCAGGGTAGAAAAACCAAAGCAAG	
J	Genotyping	CATCAGCCGCGGTGTGGATCATGCAGGCTTGG	
Hoxa5	qPCR	FWD	AAGCTGCACATTAGTCACG
		REV	CATGAGCTATTTCGATCCTT
Hoxa7	qPCR	FWD	GGCCAATTTCCGCATCTA
		REV	CGTCAGGTAGCGGTTGAAG
Hoxa9	qPCR	FWD	CTCTTCGTGTGGCAGATCAT
		REV	AGCTTCCACAATCACAATGG
Actin	qPCR	FWD	AGCTTCTTTGCAGCTCCTTCGTTG
		REV	TTCTGACCCATTCCCACCATCACA
Bcor exon9 DNA	qPCR	FWD	TGGCCGGTAGCAATTGTTGT
	qPCR	REV	GTGACAGATGACTGTGCGGA
Bcor intron6 DNA	qPCR	FWD	GCTCCACTCCTGGAGACCTA
	qPCR	REV	GCACTGAGCCTTATGGTGGT

Supplementary Table 4: list of transcription factors differentially regulated in $Bcor^{\Delta E9\text{-}10}$ *KL cells*

Cited2	Scmh1	Tfam	Hmga2	Gata1	Tal1
Etv6	Carf	Notch1	Polr3e	Tob1	
Abt1	Hcls1	Rbl1	Zfp142	Tfdp2	
Ldb1	Gata2	Nr4a1	Zfp36l1	Cited4	
Ilf3	Lmo1	Pbx1	Gcdh	Mid1	
Gtf2h1	Plagl2	Snip1	Ccnc	Nr2c1	
Cebpb	Usf2	Ptges2	Irf7	Bbx	
Mta1	Srebf1	Fosb	Lsm11	Fosl2	
Hmgb2	Gtf2a1	Relb	Zfp131	Zfp51	
Rnf141	Nfix	Elk1	Maz	Ddx54	
Btf3	Tnfaip3	Taf12	Zfp64	Hmg20a	
Mbd2	Gtf2b	Maml1	Glrp1	Clp1	
Nfkb1	Tgif2	Bcor	Homez	Baz2a	
Mcm4	Elf1	Taf6	Nr2f6	Taf7	
Tcf19	Trp53	Hdac6	Rybp	Atf6	
Pbxip1	Rfx1	Dnmt3a	Nrip1	Fos	
Sap30	Elk4	Irf5	Aebp2	Hoxa9	
Carhsp1	Runx2	Dedd2	Cops2	Lmo2	
Pbx3	Cebpg	Zdhhc21	Zhx3	Hoxa7	
Myc	Nfe2l2	Hoxa5	Sh3d19	Zfp36	

Supplementary Table 5: Statistical analysis of ChIP-seq data overlaps

**Comparison**	**Significance (p)**	**Z score**	**Direction**	**Analysis Method**
**WT KL cells – related to Fig. 3A**				
H3K27me3 regions and H2AK119ub regions	0.000999001	362.5348	Greater	regioneR[4]
H3K4me3 regions and H2AK119ub regions	0.000999001	211.194	Greater	
H3K27me3 regions and H3K4me3 regions	0.000999001	233.373	Greater	
**Overlap of genes associated with histone marks– related to Fig. 3I**	**Significance (p)**	**ODDS ratio**	**Number of overlapping genes**	**Analysis Method**
H2AK119ub associated genes and H3K27me3 associated genes	3.4E-22	15.1	29	GeneOverlap[5]
H2AK119ub associated genes and H3K4me3 associated genes	0.00015	6.7	7	
H2AK119ub associated genes and SE associated genes	0.0022	5.8	5	
H2AK119ub associated genes and DEG	0.04	2	10	
H3K27me3 associated genes and H3K4me3 associated genes	1.8E-37	15.1	51	
H3K27me3 associated genes and SE associated genes	0.55	1	4	
H3K27me3 associated genes and DEG	0.15	1.3	28	
H3K4me3 associated genes and SE	0.0065	3.8	6	
H3K4me3 associated genes and DEG	0.0017	2.2	20	
SE and DEG	0.024	1.9	14	
Upregulated Genes and Loss of ubiquitination genes in $Bcor^{\Delta E9-10}$KL cells	0.012	2.5	10	
$Bcor^{\Delta E9-10}Kras^{G12D}$ tumour KL cells: Upregulated Genes and genes associated with a Loss of ubiquitination	9E-11	6.0	23	

Supplementary References

1. Wu, D. *et al.* ROAST: rotation gene set tests for complex microarray experiments. *Bioinformatics* **26,** 2176–2182 (2010).
2. Gearhart, M. D., Corcoran, C. M., Wamstad, J. A. & Bardwell, V. J. Polycomb Group and SCF Ubiquitin Ligases Are Found in a Novel BCOR Complex That Is Recruited to BCL6 Targets. *Molecular and Cellular Biology* **26,** 6880–6889 (2006).
3. Cao, Q. *et al.* BCOR regulates myeloid cell proliferation and differentiation. *Leukemia* **30,** 1155–1165 (2016).
4. Gel, B. *et al.* regioneR: an R/Bioconductor package for the association analysis of genomic regions based on permutation tests. *Bioinformatics* **32,** 289–291 (2015).
5. Shen, L. GeneOverlap: An R package to test and visualize gene overlaps. *R Package* (2014).
