# Supplementary material for: Bcor loss perturbs myeloid differentiation and promotes leukaemogenesis
Source: Nat Commun. 2019 Mar 22;10:1347. doi: 10.1038/s41467-019-09250-6 (PMC6430802; doi:10.1038/s41467-019-09250-6)
Supplement: Supplementary file 2 — Description of Additional Supplementary Files [file 41467_2019_9250_MOESM2_ESM.pdf]

Description of Additional Supplementary Information

File Name: Supplementary Data 1
Description: Significantly differentially expressed genes (log2FC>0.5 or <-0.5, p<0.05) in BcorΔE9-10 KL cells compared with BcorWT KL cells

File Name: Supplementary Data 2
Description: Significantly differentially ubiquitinated regions (log2FC>0.3 or <-0.3, p<0.001) and associated genes in BcorΔE9-10 KL cells compared to BcorWT KL cells

File Name: Supplementary Data 3
Description: Significantly differentially expressed genes (log2FC>0.5 or <-0.5, p<0.05) in BcorΔE9-10KrasG12D KL cells compared with KrasG12D KL cells

File Name: Supplementary Data 4
Description: Significantly differentially ubiquitinated regions (log2FC>0.3 or <-0.3, p<0.001) and associated genes in BcorΔE9-10KrasG12D KL cells compared with KrasG12D KL cells

File Name: Supplementary Data 5
Description: Sequences of CRISPR guides targeting Bcor-regulated genes used for generation of a custom CRISPR library

File Name: Supplementary Data 6
Description: Statistical analysis of CRISPR dropout screen analysed with MAGeCK
